# Proximate drivers of spatial segregation in non-breeding albatrosses

**DOI:** 10.1038/srep29932

**Published:** 2016-07-21

**Authors:** Thomas A. Clay, Andrea Manica, Peter G. Ryan, Janet R. D. Silk, John P. Croxall, Louise Ireland, Richard A. Phillips

**Affiliations:** 1 British Antarctic Survey, Natural Environment Research Council, High Cross, Madingley Road, Cambridge, UK, CB3 0ET, United Kingdom; 2 Department of Zoology, University of Cambridge, Downing Street, Cambridge, CB2 3EJ, United Kingdom; 3 Percy FitzPatrick Institute, DST/NRF Centre of Excellence, University of Cape Town, Rondebosch, South Africa; 4 BirdLife International, The David Attenborough Building, Pembroke Street, Cambridge, CB2 3QZ, United Kingdom

## Abstract

Many animals partition resources to avoid competition, and in colonially-breeding species this often leads to divergent space or habitat use. During the non-breeding season, foraging constraints are relaxed, yet the patterns and drivers of segregation both between and within populations are poorly understood. We modelled habitat preference to examine how extrinsic (habitat availability and intra-specific competition) and intrinsic factors (population, sex and breeding outcome) influence the distributions of non-breeding grey-headed albatrosses *Thalassarche chrysostoma* tracked from two major populations, South Georgia (Atlantic Ocean) and the Prince Edward Islands (Indian Ocean). Spatial segregation was greater than expected, reflecting distinct seasonal differences in habitat selection and accessibility, and avoidance of intra-specific competition with local breeders. Previously failed birds segregated spatially from successful birds during summer, when they used less productive waters, suggesting a link between breeding outcome and subsequent habitat selection. In contrast, we found weak evidence of sexual segregation, which did not reflect a difference in habitat use. Our results indicate that the large-scale spatial structuring of albatross distributions results from interactions between extrinsic and intrinsic factors, with important implications for population dynamics. As habitat preferences differed substantially between colonies, populations should be considered independently when identifying critical areas for protection.

Partitioning of resources between ecologically-similar animals promotes their coexistence, and often involves the use of different areas or habitats^1^,^2^. To avoid competition, animals may exploit the same geographic space by having non-overlapping ecological niches, reflecting differences in diet, habitat or foraging behaviour^3^,^4^,^5^. Alternatively, conspecifics often segregate in geographic space, although sometimes exploit a similar habitat; however, habitat specialization may arise from the use of mutually-exclusive areas separated by distinct habitat boundaries^6^,^7^.

Colonial species such as seabirds frequently target seasonally productive areas, which can lead to intense competition among breeding birds constrained to return to land to incubate eggs or feed dependent young. Thus, seabirds are useful tools for studying the relationship between extrinsic factors such as resource availability and competition as well as intrinsic habitat preferences^6^,^8^. Birds must respond to seasonal changes in prey availability, yet they also experience differing levels of competition as breeding constraints change^6^. Additionally, factors such as body size or wing loading, energy or nutrient requirements, are important drivers of segregation^9^,^10^.

Outside the breeding period, seabirds have fewer constraints and disperse over wider areas (e.g.^11^), and so their foraging niche may be a better representation of intrinsic preferences. The recent development of miniaturized tracking devices has revealed amongst the longest known migrations on Earth, including trans-equatorial or circumpolar trips^11^,^12^,^13^. This vagility suggests few physical barriers to dispersal, yet recent evidence suggests that segregation during the non-breeding season may be a key determinant of population genetic structure^14^,^15^. In species with high movement costs such as penguins, competition and niche partitioning often leads to spatial segregation^7^,^16^; however, in more mobile species such as trans-equatorial shearwaters, there can be substantial mixing of distinct populations^12^,^17^. In addition, the few studies that investigate within-population differences indicate that non-breeding birds of different sex and breeding outcome vary in their space or habitat use, with implications for population structure and dynamics^18^,^19^.

Grey-headed albatrosses *Thalassarche chrysostoma* (hereafter GHA) forage in highly seasonal environments and often associate with frontal systems between Antarctic and sub-Antarctic waters^20^. In a pioneering tracking study, GHA from South Georgia exhibited a diversity of migration strategies; some remained resident in the southwest Atlantic, whereas others travelled to the southwest Indian Ocean, or undertook one or more circumpolar trips during a single non-breeding period^11^. As such, they are an excellent model species for investigating large-scale space use of animals over time. GHA breed every two years with a non-breeding duration of around 16 months, so deferring breeders represent a substantial proportion of the population^11^,^21^. There are major populations at seven island groups, and c. 50% of global numbers are at South Georgia^22^ (Supplementary Table S1). The non-breeding distributions of other populations are so far unknown, as are the movements of failed birds.

In this study we modelled the distributions of non-breeding GHA from different populations within a use-availability framework^8^. We compare the spatial predictions of various hypothesized drivers of space use to determine which best explained the observed patterns. This study investigates 1) the degree of spatial segregation among and within populations, 2) the influence of extrinsic factors (habitat availability and intra-specific competition) on distributions, and 3) the influence of intrinsic factors (population, sex, breeding outcome and individual) on the habitat preferences and distributions of albatrosses.

## Results

We tracked the non-breeding migrations of 66 grey-headed albatrosses from Bird Island, South Georgia (hereafter SG) and Marion Island, Prince Edward Islands (hereafter PEI), comprising 46 previously successful and 20 failed birds. The successful breeders were tracked for 490 ± 78 days (range 92–815), covering the whole non-breeding period (winter-summer-winter) for all but three birds with logger batteries that failed before retrieval. For both SG and PEI, bootstrapping analysis confirmed that our sample sizes were sufficient to make population-level inferences (Supplementary Fig. S1). We tracked 19 breeders from SG that had all failed during incubation in late December - early January (mean 25 December ± 20 d) for the subsequent 277 ± 21 days (range 242–334). As we tracked only one failed bird from PEI, this individual was excluded from our analyses. Consequently, among-population comparisons were conducted on successful individuals, and comparisons between successful and failed breeders were made on SG birds only.

### Patterns of spatial segregation

#### Among-population differences

**Figure 1:**
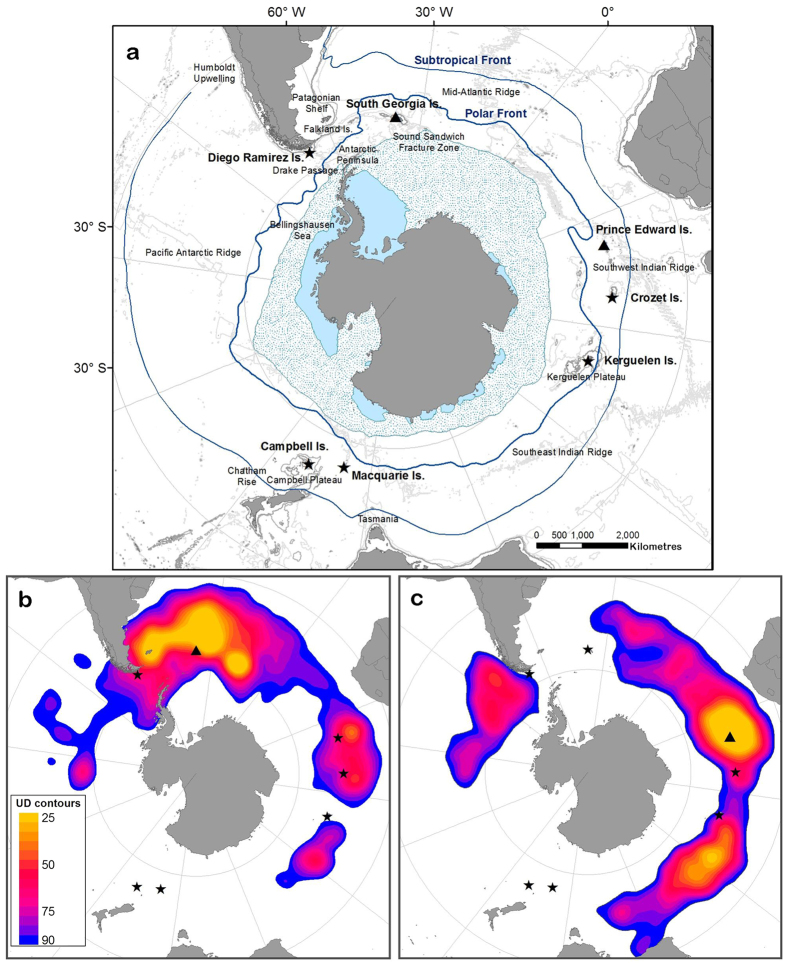
(**a**) Map of the study region including the minimum summer (light blue) and maximum winter (dotted blue) sea ice extents (>15% concentration), and 500 m, 1000 m and 3000 m isobaths. The non-breeding utilization distributions (UDs) of successful grey-headed albatrosses *Thalassarche chrysostoma* from (**b**) Bird Island, South Georgia, and (**c**) Marion Island, Prince Edward Islands. UD contours are shown in 5% intervals ranging from 25% (yellow) to 90% (dark blue). Black triangles and black stars indicate study colonies, and other breeding populations, respectively (maps produced by ArcGIS 10.1 software, http://www.arcgis.com/features).

Birds from both populations exhibited a diverse suite of migration strategies encompassing a range of oceanic habitats within the Southern Ocean (see Supplementary Material for details). Despite this high vagility, the two distributions were more spatially segregated than expected (observed overlap 0.55 and randomized overlap 1.53 ± 0.09, *P* < 0.001; Table 1), as different ocean sectors were used at different times (Supplementary Fig. S3). Successful birds from SG (n = 22) spent a large proportion of time in the southwest Atlantic; north of the colony between the Polar Front and the Subtropical Front, east of the Falkland Islands, and around the South Sandwich Fracture Zone (Fig. 1B). They also foraged around the Subtropical Front in the southwest Indian Ocean and northeast of the Kerguelen Plateau towards the Southeast Indian Ridge. In contrast, birds from PEI (n = 24) spent a large proportion of time in the Indian Ocean; around the colony towards the Southwest Indian Ridge and in the southeast Indian Ocean between the Kerguelen Plateau and Southeast Indian Ridge (Fig. 1C). They also foraged towards the Humboldt Upwelling, southeast Pacific Ocean. Spatial segregation persisted during the non-breeding summer and during both winters, but was greater in summer (observed overlap 0.40 and randomized overlap 1.35 ± 0.10, *P* < 0.001; Table 1) than winter (observed overlap 0.61 and randomized overlap 1.97 ± 0.16, *P* < 0.001; Table 1, Supplementary Fig. S2). The distribution of PEI birds tracked in 2002 and 2003 was broadly similar (observed overlap 2.16; randomized overlap 2.12 ± 0.18, *P* = 0.58), although birds in 2002 were more likely to use the southeast Pacific (Supplementary Fig. S4).

#### Within-population differences

We found no evidence of sexual segregation across the whole utilization distributions (pooled dataset UDOI method; observed overlap 1.47 and randomized overlap 1.60 ± 0.20, *P* = 0.070; Table 1); however, we did find males and females from SG segregated in their core (50%) but not general use (90%) distributions during summer only (Supplementary Table S2, Fig. S5). Females from both populations also used higher latitudes than males by c. 1°, year-round (GLMM: *Χ*^*2*^_*1*_ = 6.0, *P* = 0.014; Table 2). Males in both populations departed 6 days later than females (GLMM: *Χ*^*2*^_*1*_ = 5.1, *P* = 0.024), but did not differ in return dates (GLMM: *Χ*^*2*^_*1*_ = 2.0, *P* = 0.15). There was no evidence that males from either population travelled further than females, or varied more in their migration characteristics (Table 2).

**Figure 2:**
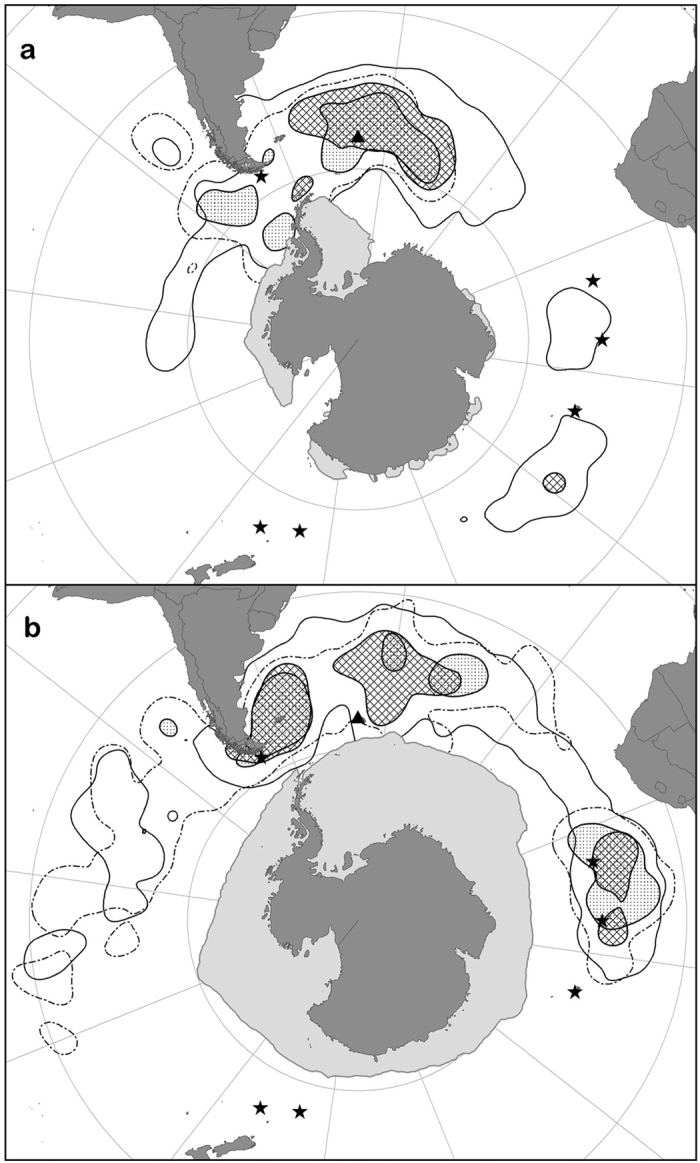
The core (50%) and general (90%) utilization distributions (UDs) of previously successful and failed grey-headed albatrosses from South Georgia, (**a**) in summer, and (**b**) in winter. Core areas are shown for failed and successful birds with hashed and dotted fill, respectively, and for general areas solid and dotted line type, respectively. The minimum summer and maximum winter sea-ice extents (<15% concentration) are shown. Black triangles and black stars indicate the study colonies, and other breeding populations, respectively (maps produced by ArcGIS 10.1 software, http://www.arcgis.com/features).

Failed birds from SG dispersed significantly less than successful birds (GLMM: *Χ*^*2*^_*1*_ = 14.9, *P* < 0.001), probably due to their shorter non-breeding period (8 *vs.* 16 months), as they covered a similar mean distance per day (GLMM: *Χ*^*2*^_*1*_ = 2.7, *P* = 0.11; see Supplementary Material for details). During summer, failed birds used more southerly areas than successful birds, and consequently, segregation was greater than expected by chance (observed overlap 1.01 and randomized overlap 1.45 ± 0.17, *P* = 0.001; Table 1, Fig. 2a). During winter, as with successful birds, many failed GHA moved east to forage in the southwest Indian Ocean (Fig. 2b); consequently, there was no apparent spatial segregation from successful birds during winter (observed overlap 1.71 and randomized overlap 1.65 ± 0.20, *P* = 0.65; Table 1). All failed GHA returned to breed the following season, but arrived slightly later (4 days) than previously successful non-breeders (GLMM: *Χ*^*2*^_*1*_ = 5.8, *P* = 0.022; Table 3).

### Drivers of spatial segregation

#### Among-population differences

For all four groups, the full model received the most support (>77% weight in each case) (Supplementary Table S7). The inclusion of tracking year improved model fit for birds from PEI during both summer (ΔAIC = −4.14) and winter (ΔAIC = −13.62), but explained a negligible proportion of model deviance (Supplementary Fig. S7). There was a large variation in performance of weekly, and individual-based cross-validation, indicating that observed distributions were predicted much better in some weeks than others, and that population-level models predict the space use of some, but not all individuals (Supplementary Tables S4, S5 and S6). Furthermore, the predictive performance (AUC) of models cross-validated between sites was always poor; during winter, PEI to SG = 0.57 and SG to PEI = 0.52; and during summer, PEI to SG = 0.52 and SG to PEI = 0.54. Consequently, we made no attempt to predict distributions of birds using models developed for the other population.

**Figure 3:**
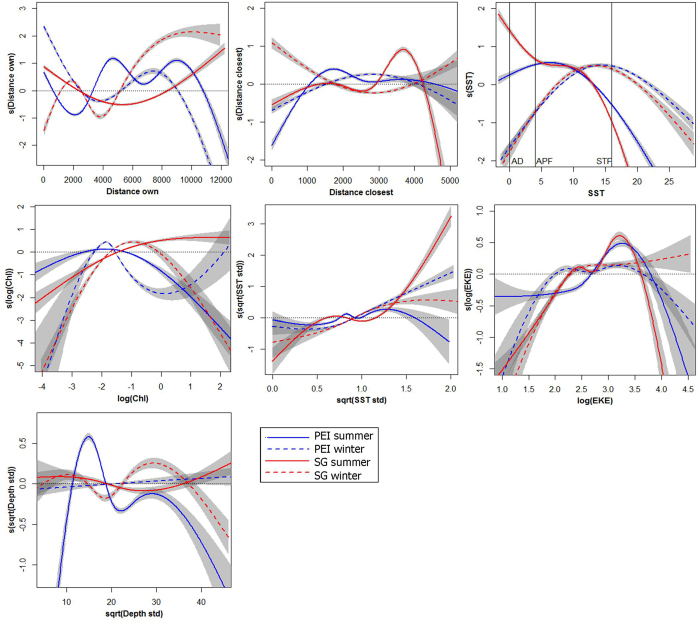
Response curves of the most important variables explaining the distribution of previously successful grey-headed albatrosses. Population is represented by colour for South Georgia (SG, in red) and the Prince Edward Islands (PEI, in blue), and season by line type for summer (solid) and winter (dashed). Standard errors of the responses from model outputs are shown in grey. Variables were classified as important when they consistently explained more than 1% of model deviance, and are shown in decreasing order of importance from top left to bottom right. Approximate values for major fronts are marked for the Antarctic Divergence (AD), Antarctic Polar Front (APF) and the Subtropical Front (STF). See Supplementary Material for more details.

Distributions of both populations were best explained by models that included habitat preferences and constraint variables associated with competition and accessibility (see Supplementary Material for details). However, the modelled responses of birds to important predictors differed with season and population. Both distance variables explained longitudinal patterns in albatross movements which were not linked directly to preferences for a particular habitat. Essentially, birds from both populations were more likely to avoid other colonies during summer than winter, and birds from PEI were more likely to avoid other colonies than birds from SG (Fig. 3).

Birds from both sites used markedly cooler waters during summer than winter; birds from SG preferred lower sea surface temperature (SST) in both seasons (Fig. 3). GHA also preferred areas with large deviations in SST, indicative of frontal regions. During summer, preferences of SG birds were associated with the Antarctic Divergence and Polar Frontal regions, along with PEI birds, and during winter, birds from both populations associated with the Subtropical Front. SG birds targeted more productive regions than PEI birds during both seasons. GHA used both a higher and wider range of chlorophyll regimes in summer than winter, and lower eddy kinetic energy; SG birds also used marginally stronger eddies than PEI birds. Finally, PEI birds preferred areas with gently-sloping bathymetry during summer, but steeper slopes during winter.

**Figure 4:**
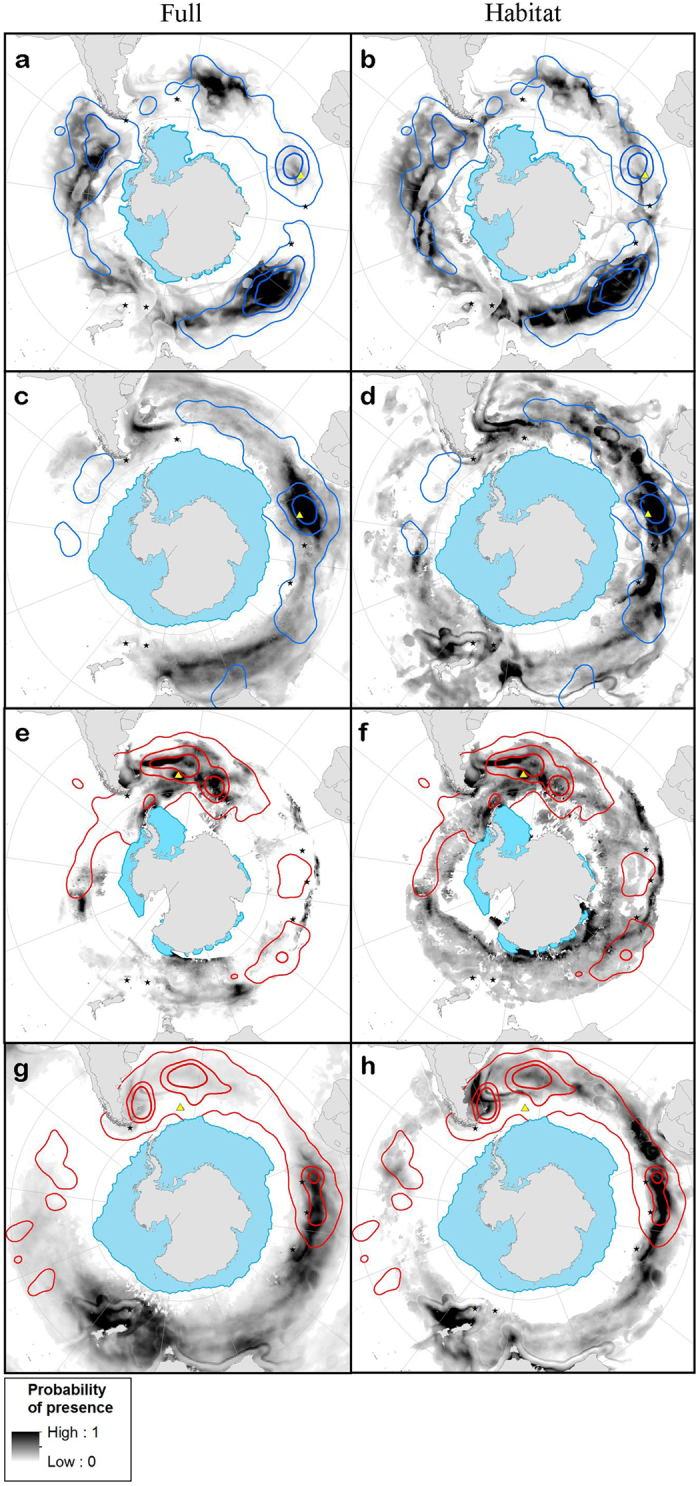
Map of the spatial predictions from the full models (left: **a, c, e, g**), and those just taking into account habitat preferences (right: **b, d, f, h**). Maps show modelled probability of presence in greyscale of birds from different populations in summer and winter; Prince Edward Islands (PEI) summer (**a, b**), and winter (**c, d**), South Georgia (SG) summer (**e, f**), winter (**g, h**). The 25%, 50% and 90% kernels of observed distributions are shown for PEI and SG birds in blue and red, respectively. The minimum summer or maximum winter sea ice extents (>15%) are shown as blue polygons and colonies of origin as yellow triangles (maps produced by ArcGIS 10.1 software, http://www.arcgis.com/features).

In slight contrast to the observed distributions, the models including only habitat variables predicted occurrences of GHA over large swathes of the Southern Ocean (Fig. 4), indicating that their distributions are limited by competition and accessibility as well as habitat preferences. Moreover, they identified areas of apparently suitable habitat that were not occupied by tracked birds. For example, during winter, both population models predicted high probability of presence east of New Zealand around the Chatham Rise, and on the Campbell Plateau, and the model for PEI predicted that those birds would use the Scotia Sea and waters around the Falkland Islands though birds were not found in these areas.

#### Within-population differences

**Figure 5:**
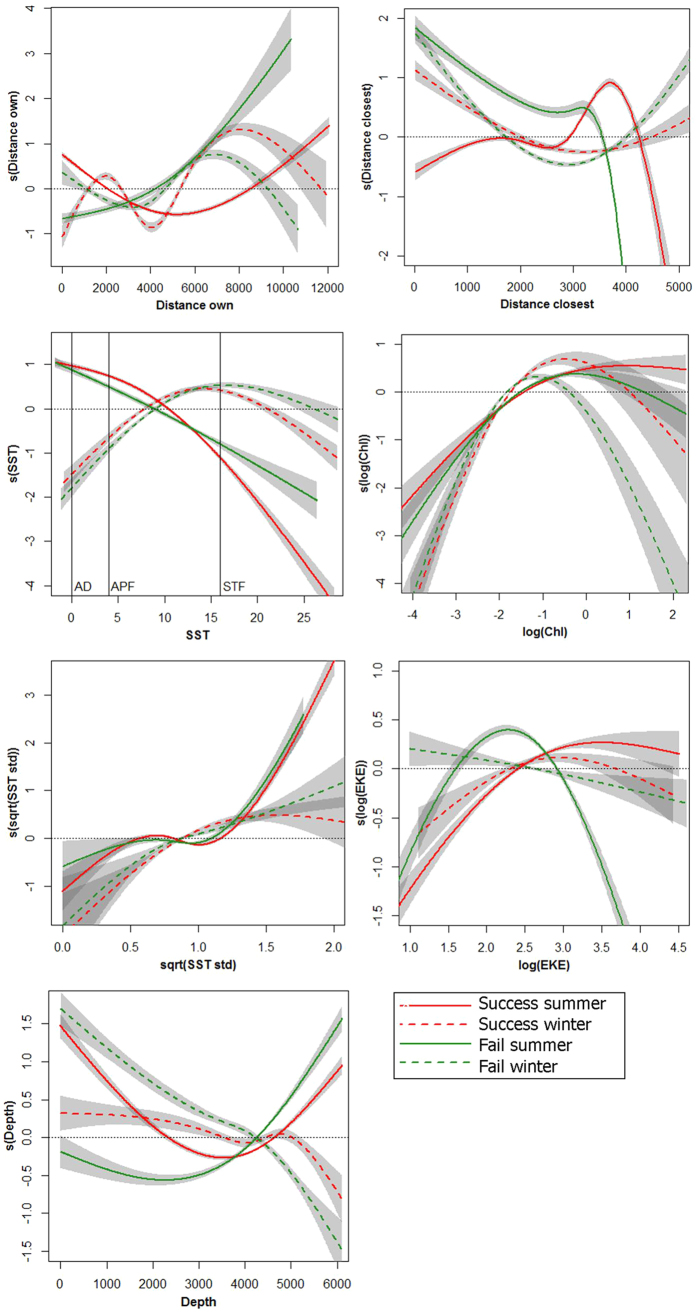
Response curves of the most important variables explaining the distribution of previously successful and failed grey-headed albatrosses from South Georgia. Breeding outcome is represented by colour for successful (red) and failed (green) birds, and season by line type for summer (solid) and winter (dashed). Standard errors of the responses from model outputs are shown in grey. Variables were classified as important when they consistently explained more than 1% of model deviance, and are shown in decreasing order of importance from top left to bottom right. Approximate values for major fronts are marked for the Antarctic Divergence (AD), Antarctic Polar Front (APF) and the Subtropical Front (STF). See Supplementary Material for more details.

We found no significant differences in model performance scores with the inclusion of sex-specific preferences, providing no evidence of sexual segregation by habitat (Supplementary Table S6). During summer, but not winter, model performance was significantly better with the inclusion of preferences associated with previous breeding outcome (Supplementary Table S5). Similar variables explained the distributions of successful and failed non-breeders. Habitat preferences of birds of different outcome corresponded more closely than those of successful birds from the two populations (Figs 3 and 4), yet there were some noticeable differences, particularly during summer. After breeding failure, birds were more likely to use areas further from SG and hence closer to other colonies, particularly around Diego Ramirez (Fig. 2), yet there was little difference during winter (Fig. 5). In both seasons, failed birds used less productive areas than successful birds. Failed birds targeted slightly colder and deeper waters in summer, and slightly warmer and shallower waters in winter. Failed birds also used frontal regions more during summer; however, they were less likely to use eddy regions than successful birds, particularly during summer.

## Discussion

In this study, we model the non-breeding distributions of a highly mobile marine predator as a function of multiple extrinsic (habitat availability and competition) and intrinsic drivers (habitat preferences, population, sex, breeding outcome). We find that patterns of spatial segregation are explained well by season-specific habitat preferences and competition at both meta-population and population scales.

It is challenging to model the distributions of pelagic seabirds as they are highly vagile and often respond to dynamic environmental cues (e.g.^23^). In particular, the Southern Ocean is a highly seasonal environment with large fluctuations in the positions of major fronts and extent of sea-ice, leading to substantial changes in productivity and prey availability^24^. Our seasonal models capture broad temporal patterns, but are unable to track the responses of albatrosses on weekly or monthly scales. Flexibility in habitat preferences of GHA likely reflects their plastic response to seasonally-changing environmental suitability. Past modelling of albatross distributions has generally assumed that animals track their environment in a fixed way (e.g.^23^,^25^); in contrast, our results emphasize that preferences may vary across long non-breeding periods, particularly for biennially-breeding species. Despite this, we find little evidence that birds from PEI tracked in different years change distribution or habitat preferences; hence, we are confident that the large-scale patterns of spatial segregation persist between years.

Whilst there is substantial evidence that spatial segregation reduces competition in breeding seabirds (e.g.^5^), few studies have concurrently modelled the importance of competition and habitat preferences on non-breeding distributions. Despite their extremely low movement costs, constraint variables associated with accessibility and intra-specific competition are important predictors of GHA distributions, particularly during summer when the density of conspecifics is likely to be much higher around the colony. Typically, birds remain at least 800–1,200 km away from other colonies, which corresponds well with the foraging ranges of breeding GHA (Supplementary Table S1). This avoidance behaviour explains why birds from PEI avoid the SG area during winter, despite similar habitat preferences. In addition, by including constraints associated with local competition, our models accurately predict the absence of both SG and PEI birds in waters around New Zealand; areas within range of the substantial breeding population on Campbell Island. Although we have not considered inter-specific competition, it is also likely to play a role in structuring albatross distributions^26^. Although, for example, congeneric black-browed (BBA) *Thalassarche melanophris* and Campbell albatrosses *T. impavida* are more specialized in targeting shelf-edges than GHA, in areas such as the Patagonian Shelf and New Zealand sub-Antarctic islands, prey depletion around colonies may be enhanced by the presence of these closely-related species^6^.

We incorporated the movement constraints of albatrosses in our null models, yet for all models *Dist. own* explained the greatest variance. Thus, despite their extreme vagility, albatrosses have preferences for areas around their own colony that are unrelated to habitat. These findings are counterintuitive, suggesting that albatrosses are willing to tolerate birds from their own population rather than exploit distant profitable patches. The costs of migration may therefore be greater than the negative interactions that might arise from local competition, or perhaps in the case of SG birds, the southwest Atlantic is productive enough to support a very large population, year-round^27^. Alternatively, birds may gain reproductive advantages by remaining close to the colony, such as additional breeding opportunities^21^.

Whilst niche partitioning can lead to spatial structuring among seabirds^3^,^4^,^28^, few studies have quantified differences in the habitat preferences of conspecifics during migration (except see^7^,^16^). Although appearing to be a habitat generalist, the greater than expected spatial segregation of our GHA populations appears to be driven predominantly by population-specific habitat preferences. Our predictive maps demonstrate the importance of niche segregation, particularly during the non-breeding summer when, for example, PEI birds appear to avoid the southwest Atlantic mainly due to habitat preferences rather than avoidance of competition (Fig. 4). Although our results contrast with a recent study comparing stable isotope ratios of albatrosses from different populations^29^, this is probably because comparisons of δ^13^C provide limited resolution of spatial overlap, particularly along longitudinal gradients. Segregation of habitat therefore appears to be the main partitioning mechanism in non-breeding albatrosses, which feed opportunistically at the surface and tend to have relatively wide dietary niches^18^,^29^. This is also supported by the broad similarity between species in diel activity patterns^30^.

Non-breeding GHA have season-specific habitat preferences related to dynamic features (frontal regions and eddies), and birds from the two populations exploit different water masses and productivity regimes. During summer, resident SG non-breeders use similar foraging areas to breeding birds^10^,^31^; colder waters around the Antarctic Peninsula, or slightly warmer waters in the Polar Frontal (PF) zone. During winter, there is considerable niche overlap with PEI birds, and predicted distributions from habitat preference-only models overlap more than in summer (Fig. 4). Both sets of birds associate with mesoscale oceanic features such as eddies. Habitat preferences of birds from PEI are more seasonally consistent, as they use waters of similar productivity throughout the year. During summer, resident birds use similar areas to breeders, reflecting strong associations with sea level anomalies created by the Agulhas Return Current to the north of the colony, and the PF to the south^32^. As GHA from both populations appear to use similar features during the breeding season and non-breeding summer, we suggest that divergent non-breeding niches may arise from local adaptation to familiar habitat during breeding^15^,^33^. Resulting specializations during the non-breeding period thus have the potential to influence dispersal patterns and ultimately the genetic structuring of seabird populations^14^,^15^.

Within a population, there is growing evidence that processes operating in one season may influence subsequent performance, known as carry-over effects^34^ (COEs). The few studies comparing the space use of seabirds of different breeding performance have found links with winter distribution^19^,^35^. In GHA, breeding outcome is linked to both space use and habitat selection, as birds that fail are more likely than sabbatical non-breeders to use sub-optimal areas (with lower productivity and mesoscale eddy activity) in the latter part of the summer. However, there appears to be no segregation between the two groups in winter prior to return to the colony.

The reasons why breeding outcome affected habitat use in summer are unclear, but could arise from variation in individual condition over short or long timescales. Failed breeders may be in better condition due to their reduced parental effort and so may withstand foraging in poorer quality habitats that are closer to other colonies than their own (Fig. 5). Alternatively, it could be that birds are less able to compete for prey with breeding birds around SG and are forced to forage nearer the breeding grounds of the major GHA population at Diego Ramirez (Supplementary Table S1). In other species, failed birds migrate further than successful birds but return to the colony at similar times, or travel less far but return sooner^19^,^35^. Yet, in biennial breeders, failed birds have less time to moult flight feathers between breeding attempts, which may be energetically costly in the following season.

In our study the link with subsequent breeding outcome is unknown, yet failed GHA return to the colony slightly later than successful birds. Late arrival at the breeding grounds can reduce reproductive success in some birds (e.g.^36^), but not others, for example the congeneric BBA^25^. Body condition appears to have greater bearing on the breeding decisions of GHA than BBA, and so COEs associated with selection of poorer habitats are likely to be more important^37^. Indeed, because GHA breed biennially, they probably have a higher cost of reproduction than annually-breeding *Thalassarche spp*.^21^. It is likely that these seasonal interactions reflect other intrinsic factors such as individual quality or breeding experience, and longitudinal studies or experimental manipulations may be necessary to fully disentangle these effects^35^,^38^.

In large, dimorphic species such as albatrosses, sex-differences in distributions have been attributed to competition, or the effects of wing-loading on flight performance^9^,^10^,^39^. In our study, although we found no evidence of sexual segregation in habitat use, females from both colonies foraged at higher latitudes, and the core areas of males from SG were segregated spatially from those of females during the summer. As males do not appear to competitively exclude females from particular habitats, the small degree of sexual segregation seems more likely to be mediated through differences in flight capabilities^10^. Our results complement previous findings that δ^13^C, but not δ^15^N, is higher in feathers of female than male GHA, suggesting the two sexes feed at similar trophic levels^18^. Although wind was not an important predictor in our models, it is important to note that finer scale differences in responses to this and other variables may be masked by the inherent error in geolocation and smaller sample sizes in some cases.

Finally, although we did not test explicitly for individual differences, in assessing model performance it was apparent that habitat preferences of some individuals differed substantially from the population mean. Similarly, stable isotope analyses indicate that GHA from Bird Island moult in a wide range of habitats, from Antarctic to subtropical latitudes, although the timing of moult is uncertain^18^. We could not test for all known sources of variation between and within populations, but are aware that other factors such as age may play a role^40^; future studies should formally test these effects.

Although predictive models are increasingly used to identify suitable habitat for poorly-known populations, there is increasing evidence that for pelagic seabirds, they have poor transferability (this study,^41^) and so caution is needed when inferring key foraging areas for untracked populations. Furthermore, our results indicate that birds from different island groups may overlap with different threats. The conservation status of GHA has recently been upgraded to Endangered by IUCN, as several populations, including SG, are in decline. In contrast, numbers at PEI appear to be stable (PG Ryan, unpublished data,^42^,^43^). Although incidental mortality in longline fisheries is believed to be a major cause of decline, GHA were more commonly recorded as bycatch in Patagonian toothfish *Dissostichus eleginoides* fisheries in the south Indian than Atlantic Ocean^44^, and so it seems unlikely to account for regional differences in population trends. On the other hand, as GHA from the two populations target different dynamic habitats, there may be contrasting impacts of climatic change. In particular, the higher breeding frequency of birds from PEI than SG^21^ suggests that environmental conditions are more benign for the former during the non-breeding period. Future research should investigate the implications that spatial and habitat segregation has on the conservation and management of this and other threatened seabird populations.

## Methods

### Logger deployment and data processing

We carried out fieldwork on two widely-separated populations; Bird Island, SG in the southwest Atlantic Ocean (54°00′ S, 38°03′ W) and Marion Island, PEI in the southwest Indian Ocean (46°54′ S, 37°45′ E). Together these archipelagos represent >60% of the global annual breeding population (Supplementary Table S1). The experimental protocol at Bird Island was approved by the Ethics Committee of British Antarctic Survey, and the study conducted in accordance with the relevant guidelines and regulations of the Government of South Georgia and the South Sandwich Islands. On Marion Island, research was conducted under permit issued by the South African Department of Environmental Affairs, following approval by the University of Cape Town’s Animal Ethics Committee. We tracked previously successful breeders from both SG (n = 22) and PEI (n = 24), and failed breeders from SG (n = 19) (see Supplementary Material for details). Handling time for deployment and retrieval of tracking devices was <10 minutes. GHA are sexually size-dimorphic^10^, and most birds were sexed from bill measurements.

We processed light data in Multitrace, providing two positions per day with a mean error of 186 km ± 114 km^45^. We excluded locations with interruptions around sunrise and sunset, and periods around the equinox (2 to 4 weeks), when latitude cannot be estimated reliably; however, individual departure and arrival times could still be derived from longitudinal movements. We created utilization distribution (UD) kernels to show spatial patterns, with the 50% and 90% UDs representing core and general use areas. To control for individual differences in tracking durations, UDs were generated for each bird and then merged so there was equal representation in our larger sample. We selected a grid size of 50 km and smoothing parameter of 200 km to account for tag error^26^,^45^.

Distributions of birds were compared in summer (mid-September to mid-May, coinciding with the breeding season) and winter (mid-May to mid-September). To determine if samples were sufficient to represent each population, a bootstrapping approach was used to randomly sample home-range area with an increasing number of individuals^46^ (see Supplementary Material for details).

### Spatial segregation

To test whether albatrosses segregate by population, sex, breeding outcome and season, we calculated the overlap between home ranges using the utilization distribution overlap index (UDOI), which is considered the most appropriate measure of overlapping space use^47^. We used a randomization procedure to determine if observed spatial segregation was greater than expected by chance; *P*-values were determined as the proportion of randomized overlaps that were smaller than the observed^48^. In order to make sure our results were repeatable across overlap indices, we compared results using the UDOI method with that of the home-range method (PHR) which measures the proportion of overlap of home-ranges at certain isopleths, in our case the 50% and 90% isopleths^47^ (see Supplementary Material for details). For ease of interpretation, only results from the UDOI method are reported in the main text (see Supplementary Table S2 for PHR results).

### Habitat Modelling

We used a habitat preference modelling approach to quantify space use as a function of available habitat, by comparing where an animal was observed (tracking location) with where it could have gone (pseudo-absence), if it had no particular preference^49^. We simulated tracks, accounting for availability of suitable habitat by incorporating individual movement constraints, which is particularly important for GHA given the large variability in migration strategies^11^. Simulations for each individual were correlated random walks (CRW) generated in the R package adehabitatLT^50^ using two movement parameters: step length and turning angle. We tested the effects of different numbers of simulations on model performance^25^ (see Supplementary Material for details).

As animals rarely respond to the environment in a linear way^49^, we used a generalized additive modelling (GAM) approach with smooth splines for environmental predictors and a binomial error structure^51^. We selected static and dynamic variables known to be important for albatrosses^20^,^31^,^32^ (Supplementary Table S3), including 1) sea surface temperature (*SST*) and 2) gradient as measured by the standard deviation of *SST* (*SST std*), indicative of water mass and frontal regions; 3) chlorophyll α concentration (*Chl*) as a proxy of ocean productivity; 4) ocean floor depth (*Depth*) and 5) slope, represented by the standard deviation of *Depth* (*Depth std*), to indicate productive bathymetric areas such as shelf-breaks, seamounts and upwelling; 6) sea-level anomaly (*SLA*) and 7) eddy kinetic energy (*EKE*) as indicators of mesoscale oceanography associated with currents and eddies; and 8) wind speed (*Wind*) as albatrosses are known to be constrained by high flight costs^52^. We also incorporated; 9) minimum distance to the colony (*Dist. own*) and 10) minimum distance to the closest other colony (*Dist. closest*) to determine the importance of habitat accessibility and intra-specific competition, which limit distributions of breeding albatrosses^6^. Oceanographic variables were sampled within a temporal window of each location in observed and simulated tracks (see Supplementary Material for details).

We constructed separate models for different classes of birds because of computational demands and difficulties of interpreting high order interactions. Initial models testing for interactions between population and season were significant, so we split the model into four components, by population (SG vs. PEI) and season (summer vs. winter). For each model, we ran all possible combinations of predictors and calculated AIC values using the dredge function in the R package MuMIn^53^. Candidate models were ranked according to AIC and weight. We then individually assessed the importance of variables based on proportion of deviance explained (see Supplementary Material for details). For PEI models we included the tracking year as a fixed effect to test for annual differences in distributions, then compared models with and without year and selected those with the lowest AIC values.

To identify the main extrinsic drivers, we constructed models representing only habitat (habitat variables), the constraints associated with competition and accessibility (distance variables), and the full model (all variables). We used area under the receiver operator characteristic curve (AUC) to evaluate performance of models on a weekly basis, in the PresenceAbsence package in R^54^. Values of 0.5–0.7, 0.7–0.9, and >0.9 represent poor, reasonable and very good model performance, respectively. We created spatial predictions for each population in summer and winter using the cut-off of highest sensitivity and lowest proportion of false positives along the ROC curve to distinguish suitable and unsuitable habitat. We compared weekly AUC scores of the three predictor types to determine which drivers (full, habitat or distance constraints) best matched observed spatial patterns (see Supplementary Material). We also tested transferability of population-specific models across sites by cross-validation using AUC scores, whereby each individual was a data fold. Iteratively, each model was trained on all-but-one fold and tested on the remaining one, withholding each fold in turn. Metrics of model performance thus took individual differences into account.

To test for intrinsic influences of sex and breeding outcome (failed or successful) on space use, we ran models with and without these variables as smoothers. To assess sex differences, we used the original four population models (above), and to assess breeding outcome differences, used the pooled dataset of failed and successful birds from South Georgia during summer and winter. We tested model performance on each individual, as above, comparing the resulting AUC scores of models with and without sex- and breeding outcome-specific smoothers. All analyses were conducted in the software R v. 3.1.1^55^. Unless otherwise indicated, values are presented as mean ± SD.

## Additional Information

**How to cite this article**: Clay, T. A. *et al*. Proximate drivers of spatial segregation in non-breeding albatrosses. *Sci. Rep.* **6**, 29932; doi: 10.1038/srep29932 (2016).

## Supplementary Material

Supplementary Information

## Figures and Tables

**Table 1:** Observed and randomized overlap (Utilization distribution overlap index, UDOI) of utilization distributions (UD) between different groups of grey-headed albatrosses; population (South Georgia or SG and Prince Edward Islands or PEI) by season, sex by population and season, and breeding outcome by season.

Class	Observed	Randomized	*P*	
Population (All successful)				
Summer	0.40	1.35 ± 0.10	**<0.001**	
Winter	0.61	1.97 ± 0.16	**<0.001**	
Sex (All successful)				
PEI Summer	1.49	1.52 ± 0.16	0.35	
PEI Winter	2.18	2.58 ± 0.29	0.074	
SG Summer	1.33	1.32 ± 0.24	0.50	
SG Winter	1.31	1.16 ± 0.28	0.72	
Breeding outcome (All SG)				
SG Summer	1.01	1.45 ± 0.17	**0.001**	
SG Winter	1.71	1.65 ± 0.20	0.61	

Breeding outcome comparisons are for SG only. Randomized overlaps are shown as a mean ± SD and *P* represents the proportion of randomized overlaps that were smaller than the observed. For more information see Supplementary Methods. Significant differences are shown in bold.

**Table 2:** Comparison of migration characteristics (mean ± SD) of grey-headed albatrosses from Bird Island, South Georgia (SG) and Marion Island, Prince Edward Islands (PEI) by population and by sex.

	Population				Sex			
	*P*	*Χ*^*2*^	SG	PEI	*P*	*Χ*^*2*^	Female	Male
Departure date	*0.17*	*1.9*	9 May (±10)	6 May (±9)	***0.024***	*5.1*	5 May (±10)	11 May (±7)
Return date	***0.002***	*9.9*	23 Sept. (±6)	3 Sept. (±6)	*0.15*	*2.0*	16 Sept. (±12)	9 Sept. (±10)
Non-breeding period (days)	***0.003***	*8.6*	502 (±12)	486 (±10)	***0.005***	*7.9*	499 (±13)	487 (±10)
Proportion circumpolar (%)	*0.06*	*3.5*	52 (±51)	83 (±38)	*0.89*	<*0.1*	67 (±48)	72 (±46)
Cumulative distance (km)	*0.27*	*1.2*	161,000 (±26,000)	173,000 (±21,000)	*0.87*	<*0.1*	166,000 (±27,000)	168,000 (±20,000)
Mean distance/day (km)	*0.46*	*0.5*	381 (±55)	398 (±44)	*0.72*	*0.1*	386 (±54)	397 (±44)
Maximum range (km)	***0.037***	*4.4*	6,700 (±2,900)	8,200 (±1,200)	*0.86*	<*0.1*	7,400 (±2,500)	7,600 (±1,900)
Mean longitude (°)	***<0.001***	*11.3*	−19.1 (±21.9)	33.7 (±20.2)	*0.29*	*1.1*	1.8 (±35.3)	20.0 (±28.9)
Mean latitude (°)	***0.019***	*5.5*	−48.5 (±1.6)	−47.1 (±1.6)	***0.014***	*6.0*	−47.4 (±1.7)	−48.2 (±1.8)
Area 50% UD (1,000,000 km^2^)*	*0.77*	*0.1*	2.9 (±1.1)	2.9 (±1.1)	***0.028***	*4.8*	3.2 (±1.2)	2.5 (±0.8)
Area 90% UD (1,000,000 km^2^)*	*0.37*	*0.8*	14.0 (±7.1)	15.1 (±5.0)	*0.28*	*1.2*	15.3 (±6.5)	13.4 (±5.2)

*P*-values are the result of GLMMs of population and sex. Observed means are given and have been calculated from the pooled dataset. For more information see Supplementary Methods. Proportion circumpolar represents the percentage of birds that performed circumpolar trips. Significant differences are shown in bold.

^*^Average area of UD for individual birds.

**Table 3:** Non-breeding characteristics (mean ± SD) of previously successful and failed grey-headed albatrosses from Bird Island, South Georgia.

	Breeding outcome			
	*P*	*Test*	Successful	Failed
Departure date	***<0.001***	*1733*	9^th^ May (±10)	25^th^ Dec. (±20)
Return date	***0.022***	*5.8*	23^rd^ Sept. (±6)	27^rd^ Sept. (±7)
Non-breeding period (days)	***<0.001***	*1489*	502 (±12)	277 (±21)
Proportion circumpolar (%)	***<0.001***	*14.9*	52 (±51)	5 (±22)
Cumulative distance (km)	***<0.001***	*89.7*	161,000 (±26,000)	82,000 (±14,000)
Mean distance/day (km)	*0.11*	*2.7*	381 (±55)	354 (±41)
Maximum range (km)	*0.07*	*3.5*	6,700 (±2,900)	5,300 (±1,600)
Mean longitude (°)	*0.06*	*3.7*	−19.1 (±21.9)	−33.3 (±24.6)
Mean latitude (°)	*0.26*	*1.3*	−48.5 (±1.6)	−49.9 (±3.5)
Area 50% UD (1,000,000 km^2^)*	***0.019***	*6.1*	2.9 (±1.1)	2.0 (±0.6)
Area 90% UD (1,000,000 km^2^)*	***0.007***	*8.4*	14.0 (±7.1)	7.6 (±2.3)

*P*-values are from GLMs including breeding outcome, sex and their two-way interaction. The interaction and the effect of sex were not significant in all cases and are not shown. The test statistics depended on the error structure of the model (see Supplementary Methods). Proportion circumpolar represents the percentage of birds that performed circumpolar trips. Significant differences are shown in bold.

^*^Average area of UD for individual birds.
