# Supplementary material for: Proximate drivers of spatial segregation in non-breeding albatrosses
Source: Sci Rep. 2016 Jul 21;6:29932. doi: 10.1038/srep29932 (PMC4956756; doi:10.1038/srep29932)
Supplement: Supplementary Information [file srep29932-s1.pdf]

Supplementary Information

Proximate drivers of spatial segregation in non-breeding albatrosses

Thomas A. Clay[1, 2*], Andrea Manica[2], Peter G. Ryan[3], Janet R. D. Silk[1], John P. Croxall[1, 4], Louise Ireland[1] and Richard A. Phillips[1]

[1] British Antarctic Survey, Natural Environment Research Council, High Cross, Madingley Road, Cambridge, UK, CB3 0ET, United Kingdom

[2] Department of Zoology, University of Cambridge, Downing Street, Cambridge, CB2 3EJ, United Kingdom

[3] Percy FitzPatrick Institute, DST/NRF Centre of Excellence, University of Cape Town, Rondebosch, South Africa

[4] BirdLife International, The David Attenborough Building, Pembroke Street, Cambridge, CB2 3QZ, United Kingdom

*Corresponding author: tommy.clay@outlook.com

Methods

Deployment details and spatial analyses

We deployed 47 geolocators (British Antarctic Survey, Cambridge, UK) on chick-rearing adults at Bird Island in April 1999, all of which fledged chicks, and we retrieved and downloaded data from 22 loggers (from 6 males, 16 females) in September - November 2000 (Croxall et al. 2005). In November 1999, we deployed 29 loggers on adults, all of which failed during late incubation in December and January, and recovered 19 the following breeding season (from 9 males, 5 females, 5 unsexed). On Marion Island, we deployed 20 and 18 geolocators in April 2002 and 2003, of which we recovered 14 and 11 devices in October 2003 and 2004, respectively (total 12 males, 12 females). All but one bird at PEI fledged a chick; data from this failed bird were excluded from analyses for sample size considerations.

For comparison of utilization distributions (UDs), we selected the 90% rather than the 95% contour to remove locations associated with transient migratory phases. The bootstrapping of sample size was for the 50% and 90% UDs and we repeated this for 1,000 iterations. It was assumed that when home-range area reached an asymptote, a sufficient number of individuals had been tracked. We ran a series of models to compare the non-breeding characteristics of birds from different groups. We compared the combined effect of population and sex for successful birds with generalized linear mixed-effects models (GLMMs) using the lme4 package in R[1]. We used a Gaussian error structure for all models except one, where a binomial structure was used as the response was a proportion (percentage circumpolar trips). The random effect of year was included to control for potential differences between the two years of tracking data for PEI. We included sex, population and their two-way interaction as explanatory covariates and used backwards model selection to assess important variables. In all models, the interaction between sex and population was non-significant (*P*-values are not reported) (Table

2). We also carried out a similar procedure with breeding outcome for SG birds, using generalized linear models (GLMs) to test for the effects of sex, breeding outcome and their two-way interaction. *F*-test values are quoted for most characteristics, and a Chi-square test for the remainder (Table 3). None of the two-way interactions between breeding outcome and sex or any of the sex effects were significant, thus only the influence of breeding outcome is shown (Table 3).

The null hypothesis of our randomization procedure was that there would be no difference in the spatial distribution of the two groups. If upheld, then the size of the overlap should not be significantly different from the size of the overlap if the composition of the groups were randomly assigned[2]. We generated our null expectation by creating kernels from groups that had been assigned randomly using the same sample sizes as the original groups and calculated the overlap. We calculated our randomizations 1000 times from the pooled dataset. *P*-values were determined by the proportion of random overlaps that were smaller than the observed overlap; i.e. if the observed overlap was less than all randomizations, then $P \leq 0.001$[2]. Firstly, we calculated spatial segregation for PEI birds from both tracking years to determine if the distributions were significantly different; this was not the case, and years were pooled for further analyses.

Habitat modelling

We modelled simulated movements for our null model in the form of correlated random walks[3] (CRW). Each CRW was assigned to a real bird track on a given date. We removed the first and last two weeks of the non-breeding season, which were likely to correspond with periods of directed, migratory movements, as these are more challenging to integrate into the CRW approach. We also restricted our simulations to not occur over land, and to be confined within the range of all the locations, defined by the local convex hull non-parametric kernel[4].

The range was expanded by a 200 km buffer to take into account the mean error of geolocation[5]. Using methods similar to Žydelis *et al.* (2011), we tested the effects of different numbers of simulations on the performance of our models[6]. We ran a set of models consisting of all observed tracks and varying numbers of simulations of up to 50 per individual[6]. We found that both the χ^2 values for each parameter and the area under receiver operator curve (AUC) stabilized at about 20-30 simulations per individual (Fig. S6). Consequently, we chose 30 simulations per observed track for each of our models.

We sourced our habitat variables from online databases (see Table S3). Ocean floor depth was sampled from the GEBCO bathymetric dataset[7]. Monthly composites of sea surface temperature (*SST*) and chlorophyll α concentrations (*Chl*) were extracted to avoid data loss due to cloud cover. *SST* was downloaded from the NOAA Pathfinder Advanced Very High Resolution Radiometer (AVHRR) *v.5* dataset, *Chl* from the SeaWiFS sensor, made available from the Oceancolor website (http://oceancolor.gsfc.nasa.gov/). Both sea level anomaly (*SLA*) and eddy kinetic energy (*EKE*) were used as indicators of mesoscale turbulence[8]. We considered *EKE* as a measure of the intensity of mesoscale activity, and *SLA* as a measure of the eddy type (warm or cold). Eight-day *SLA* was taken from the Delayed-Time MLSA updated dataset and *EKE* was calculated from geostrophic current velocities. Both were extracted from Aviso (http://www.aviso.oceanobs.com/) via the Marine Geospatial Ecology Tools (MGET) extension in ArcGIS 10.1[9]. Wind speed was downloaded from Ifremer (http://cersat.ifremer.fr/) at an 8-day temporal resolution. Up until August 1999, only ERS-2 gridded means were available, after which we used QuikSCAT gridded mean wind fields. We used the standard deviation of the values within each buffer as a measure of the gradient in bathymetry (*Depth std*) to represent shelf-edges or seamounts, and also for sea surface temperature (*SST std*) and productivity (*Chl std*) as GHA are known to target frontal systems[10,11].

We investigated appropriate measures of competition and accessibility in the form of two groups of candidate predictor variables: 1) the projected distance from the population of origin and from the closest major population, and 2) this distance weighted by population size[12]. Distances were calculated in the South Pole Lambert Azimuthal Equal Area projection in ArcGIS 10.1 using the *cost distance* function and were not allowed to cross land barriers. As both sets of distance and weighted distance variables were strongly correlated, we ran preliminary models containing either distance or weighted distance variables. In both cases, the distance variables produced models with lower AIC scores. Consequently, we consider the distance from the nearest major population as an indicator of avoidance due to competition, and the distance from population of origin as an indicator of potential avoidance of local birds or accessibility of a habitat, depending on the response. Major populations were considered to be island groups with >50 annual breeding pairs (Table S1).

To account for geolocation error[5], we created a 200 km buffer around each location and simulation, and extracted the mean value within each buffer. All records with incomplete environmental information e.g. due to cloud cover, were removed before analysis, which, for the complete dataset summed to around 20% of all records. We standardized variables to improve the spread of the data and model convergence; *Chl* and *EKE* were log-transformed and *SST std* and *Depth std* were square root-transformed. We checked for correlation of predictor variables by calculating all pairwise Spearman rank correlation coefficients. When pairs of predictor variables were highly correlated (>0.6), we ran two models with each predictor and selected the model with the lowest Akaike Information Criteria (AIC) value[13]. *Chl* and *Chl std* were highly correlated and so *Chl* was chosen as it resulted in greatest model fit.

The inclusion of a random intercept for individual ID can help control for variability in response to the environment[14,15]; however, model selection and inference in large datasets

is computationally demanding within the mixed effects framework. Thus, we chose to use generalized additive models (GAMs), and tested the trained models on each individual, so that metrics of model performance took individual differences into account. When constructing our GAMs, we initially set the maximum number of knots to 4 to reduce over-fitting and increased the number of knots only if the model response curves did not match the raw data. Also, to reduce over-parameterization, smoothers were produced using cubic regression splines with shrinkage which penalize variables during fitting[14]. We checked for spatial autocorrelation of our model residuals using semi-variograms in the Geo-R package in R[16]; this was not detected at the relevant spatial scale and was therefore deemed not to influence the results.

We ran all combinations of variables using the dredge function and ranked models according to AIC, and individually assessed the importance of each variable based on the proportion of deviance explained. For each term, we calculated the unique deviance explained by subtracting the deviance of the model excluding that term from the full model deviance. We also calculated the deviance shared with other predictors by subtracting this unique deviance from the amount explained by a model just with that term.

We created spatial predictions at the temporal scale of the underlying environmental data; monthly for *Chl, SST* and *SST std*, and weekly for the remaining dynamic variables. The environmental data were also resampled to match the spatial resolution of the original extractions. Weekly predictions of suitable habitat were scaled to 1 and summed over the season to produce spatial predictions for each population in summer and winter. We compared weekly AUC scores of the three predictor types using linear models, with AUC score as the response, and the week and predictor type (full, habitat or constraints) as covariates. If the best model retained the predictor type, we concluded those drivers were better at explaining spatial patterns. We ran paired t-tests to determine if the inclusion of the

sex or breeding outcome-specific smoother produced significantly different AUC scores. If significant, this confirmed that the relevant factor influenced habitat use.

Results

Migration characteristics

A greater proportion of birds from Prince Edward Islands (PEI) tended to perform circumpolar trips (83%) than South Georgia (SG) birds (52%) (GLMM: $X^2_1 = 3.5$, $P = 0.06$; Table 2); leading birds from PEI to range further from the colony (8,200 ± 1,200 km to 6,700 ± 2,900 km; GLMM: $X^2_1 = 4.4$, $P = 0.037$). Despite travelling further, the non-breeding period was shorter for PEI birds (GLMM: $X^2_1 = 8.6$, $P = 0.003$), principally because they returned 19 days earlier to the colony (GLMM: $X^2_1 = 9.9$, $P = 0.002$).

After breeding failure, SG birds (58%) travelled west to the southeast Pacific, and all but one of the remainder (37%) stayed in the local area (Fig. 2). One bird commenced a remarkable clockwise circumpolar trip, making a direct commute of over 18,000 km to the central Pacific in just over 17 days. During mid-summer, failed birds used southerly areas around the Bellingshausen Sea and then moved further north to the west coast of Chile during late-summer (Fig. 2). As a result, failed and successful birds were more segregated than expected by chance (observed overlap 1.01 and randomized overlap 1.45 ± 0.17, $P = 0.001$; Table 1). During winter, like the successful breeders, failed GHA that went east foraged in the southwest Indian Ocean, whereas local birds mainly foraged around the Falkland Islands with a few travelling to the Mid-Atlantic Ridge in late-winter (Fig. 2). As a result, there was no apparent spatial segregation from successful breeders during winter (observed overlap 1.71 and randomized overlap 1.63 ± 0.22, $P = 0.65$).

Habitat modelling

We document details on variable importance and responses to important predictors in this section. *Dist. own* was consistently the most important predictor, explaining a large proportion of unique deviance (Fig. S7). Similarly, *Dist. closest*, *SST* and *Chl* (except PEI summer) were important for both populations in summer and winter, whereas *SST std.* was important during winter. *EKE* and *Depth std.* were more important for birds from PEI during both summer and winter. Although included in models, *SLA* explained a negligible proportion of model deviance, and *Wind speed* and *Depth* were also largely unimportant. The variables *Dist. own* and *Dist. closest* explained longitudinal patterns in albatross movements (Fig. S8) which were not linked to preferences for a particular habitat. Albatross presence declined with increasing distance to around 500 – 1000 km from the colony for PEI birds during both seasons and for SG birds during summer (Fig. 3). The opposite pattern was observed for the *Dist. closest* variable. The exception was for SG birds during winter, which were more likely to use areas away from their colony and nearer to other colonies. This relationship was most likely driven by the lower density of SG birds around their colony during this period (Fig. S2). With regards to model predictions, as expected, the constraint-only models predicted distributions that were clustered in particular regions (Fig. S8). These variables accurately centered distributions around the colony, or occasionally further away; however, some predictions were spurious, including under-prediction of core areas around the colony or over-prediction in other regions.

When comparing birds of different breeding outcome *Dist. Own*, *Dist. closest*, *SST* and *SST std* explained a large proportion of model deviance in winter, while in summer, *Dist. closest* and *SST* were important for successful and failed birds (Fig. S7). *Chl* was less, and *Depth* more important for failed birds during both seasons.

References

1. Bates, D. *et al. Lme4: Linear Mixed-Effects Models using "Eigen" and S4* (2015).
2. Breed, G. A., Bowen, W. D., McMillan, J. I. & Leonard, M. L. Sexual segregation of seasonal foraging habitats in a non-migratory marine mammal. *Proc. R. Soc. B* **273**, 2319–2326 (2006).
3. Kareiva, P. M. & Shigesada, N. Analyzing insect movement as a correlated random walk. *Oecologia* **56**, 234–238 (1983).
4. Getz, W. M. *et al.* LoCoH: nonparameteric kernel methods for constructing home ranges and utilization distributions. *PLoS ONE* **2**, e207 (2007).
5. Phillips, R. A., Silk, J. R. D., Croxall, J. P., Afanasyev, V. & Briggs, D. R. Accuracy of geolocation estimates for flying seabirds. *Mar. Ecol. Progr. Ser.* **266**, 265–272 (2004a).
6. Žydelis, R. *et al.* Dynamic habitat models: using telemetry data to project fisheries bycatch. *Proc. R. Soc. B* **278**, 3191–3200 (2011).
7. Intergovernmental Oceanographic Commission [IOC] *Centenary edition of the GEBCO Digital Atlas*. Intergovernmental Oceanographic Commission and the International Hydrographic Organization as part of the General Bathymetric Chart of the Oceans. British Oceanographic Data Centre, Liverpool, UK [CD-ROM.] (2003).
8. Wakefield, E. D., Phillips, R. A. & Matthiopoulos, J. Quantifying habitat use and preferences of pelagic seabirds using individual movement data: a review. *Mar. Ecol. Progr. Ser.* **391**, 165–182 (2009).
9. Roberts, J. J., Best, B. D., Dunn, D. C., Treml, E. A. & Halpin, P. N. Marine Geospatial Ecology Tools: An integrated framework for ecological geoprocessing

with ArcGIS, Python, R, MATLAB, and C++. *Environ. Modell. Soft.* **25**, 1197–1207 (2010).

10. Waugh, S. M. *et al.* Exploitation of the marine environment by two sympatric albatrosses in the Pacific Southern Ocean. *Mar. Ecol. Progr. Ser.* **177**, 243–254 (1999).
11. Nel, D. C. *et al.* Exploitation of mesoscale oceanographic features by grey-headed albatross *Thalassarche chrysostoma* in the southern Indian Ocean. *Mar. Ecol. Prog. Ser.* **217**, 15-26 (2001).
12. Wakefield, E. D. *et al.* Habitat preference, accessibility, and competition limit the global distribution of breeding black-browed albatrosses. *Ecol. Monogr.* **81**, 141–167 (2011).
13. Burnham, K. P. & Anderson, D. R. *Model Selection and Multimodel Inference* (Springer, 2004).
14. Wood, S. *Generalized Additive Models: An Introduction with R* (CRC Press, 2006).
15. Aarts, G., MacKenzie, M., McConnell, B., Fedak, M. & Matthiopoulos, J. Estimating space-use and habitat preference from wildlife telemetry data. *Ecography* **31**, 140–160 (2008).
16. Diggle, P. J. *GeoR: Analysis of Geostatistical Data* (2015).
17. Poncet, S. *et al.* Status and distribution of wandering, black-browed and grey-headed albatrosses breeding at South Georgia. *Polar Biol*. **29**, 772–781 (2006).
18. Ryan, P. G., Jones, M. G. W., Dyer, B. M., Upfold, L & Crawford, R. J. M. Recent population estimates and trends in numbers of albatrosses and giant petrels breeding at the sub-Antarctic Prince Edward Islands. *Afr. J. Marine Sci*. **31**, 409-417 (2009).

19. Weimerskirch, H., Bartle, J. A., Jouventin, P. & Stahl, J. C. Foraging ranges and partitioning of feeding zones in three species of southern albatrosses. *Condor* **90**, 214–219 (1988).

20. Moore, P. J. Abundance and population trends of mollymawks on Campbell Island in *Science for Conservation* 242, 62 (2004).

21. Robertson, G. *et al.* An estimate of the population sizes of black-browed (*Thalassarche melanophrys*) and grey-headed (*T. chrysostoma*) albatrosses breeding in the Diego Ramírez Archipelago, Chile. *Emu* **107**, 239–244 (2007).

22. Phillips, R. A., Silk, J .R. D., Phalan, B., Catry, P. & Croxall, J.P. Seasonal sexual segregation in two *Thalassarche* albatross species: competitive exclusion, reproductive role specialization or foraging niche divergence? *Proc. R. Soc. B* **271**, 1283–91 (2004b).

23. Catry, P., Phillips, R. A., Phalan, B., Silk, J. R. D. & Croxall, J. P. Foraging strategies of grey-headed albatrosses *Thalassarche chrysostoma*: integration of movements, activity and feeding events. *Mar. Ecol. Prog. Ser.* **280**, 261–273 (2004).

24. Nel, D. C. *et al.* Foraging ecology of grey-headed mollymawks at Marion Island, southern Indian Ocean, in relation to longline fishing activity. *Biol. Conserv.* **96**, 219–231 (2000).

25. Terauds, A., Gales, R., Baker, G. B. & Alderman, R. Foraging areas of black-browed and grey-headed albatrosses breeding on Macquarie Island in relation to marine protected areas. *Aquat. Conserv.* **16**, 133–146 (2006).

26. Robertson, G. *et al.* Black-browed albatross numbers in Chile increase in response to reduced mortality in fisheries. *Biol. Conserv.* **16**, 319–333 (2014).

Table S1. Summary of global breeding populations of grey-headed albatrosses and their foraging ranges during breeding.

Population and abbreviation	Annual breeding population (pairs)	Latitude (°)	Distance and direction of PF from colony (km)	Mean max. foraging range (km)		Absolute max. foraging range (km)	
				Inc.	CR	Inc.	CR
South Georgia (SG)	47,674 [1]	-54.0	390 N	1372 [6]*	571 [6]*	-	1,760 [7]
Prince Edward Is. (PEI)	10,344 [2]	-46.8	337 SW	2182 ±1,408 [8]	722 ± 538 [8]	4,060 [8]	1,812 [8]
Crozet Is. (CRO)	5,940	-46.1	673 S	-	-	-	-
Kerguelen Is. (KER)	7,900 [3]	-49.4	191 N	-	-	-	-
Macquarie Is. (MAC)	94	-54.6	340 S	-	-	*c.* 2,100 [9]¥	-
Campbell Is. (CAM)	6,600 [4]	-52.5	857 S	-	1,567 ±751 [10]	-	2,714 [10]
Diego Ramirez Is. (DR)	17,178 [5]	-56.5	366 S	-	-	*c.* 3,100 [11]¥	*c.*1,700 [11]¥

[1]Poncet et al. 2006[17], [2]Ryan et al. 2009[18], [3]Weimerskirch et al. 1988[19], [4]Moore 2004[20], [5]Robertson et al. 2007[21], [6]Phillips et al. 2004[22], [7]Catry et al. 2004[23], [8]Nel et al. 2000[24], [9]Terauds et al. 2006[25], [10]Waugh et al. 1999[10], [11]Robertson et al. 2014[26]. *Standard deviations not available as means calculated from averages. ¥ Values estimated from published figure of maximum foraging areas during breeding. PF = Polar Front, Inc. = incubation, CR = chick-rearing. Mean maximum foraging range represents the mean of individuals and absolute maximum represents the largest individual foraging range. Values are mean ± standard deviation.

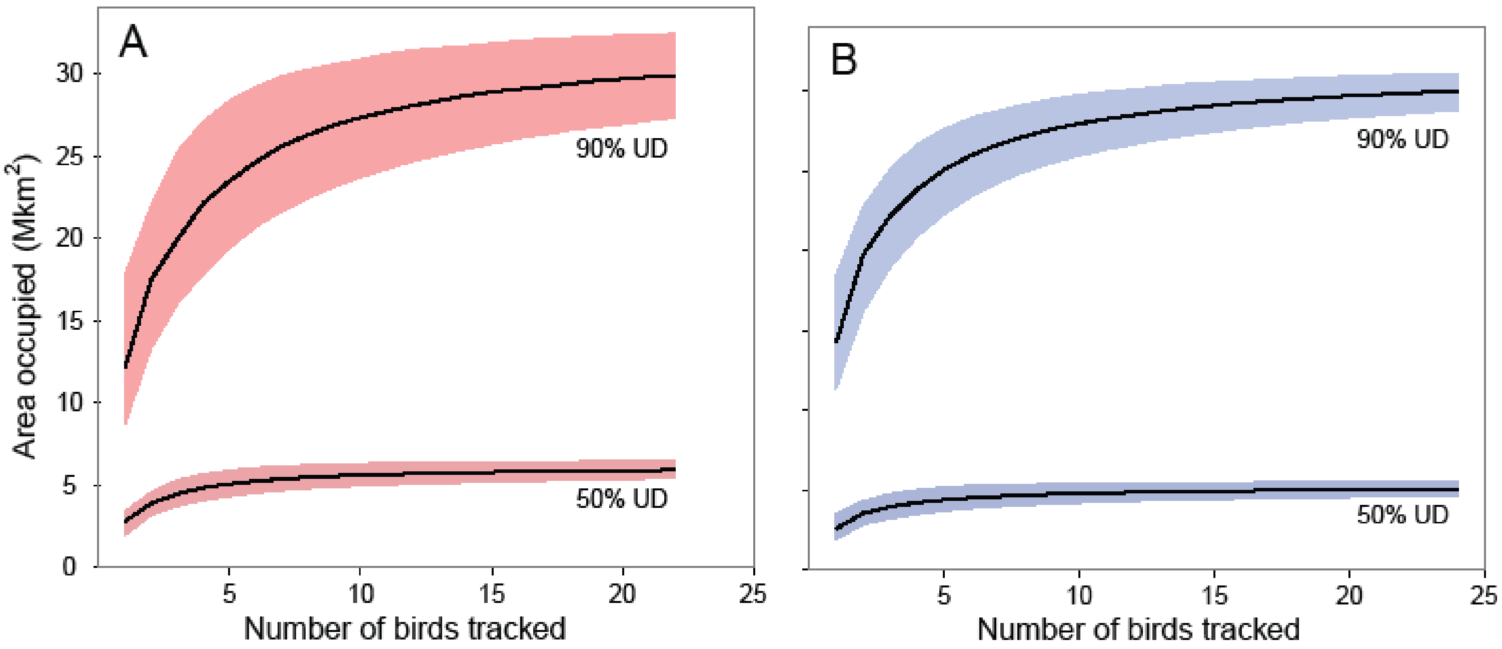

Figure S1. Bootstrapped area of 50% and 90% utilization distributions (UDs) of successful non-breeding grey-headed albatrosses relative to sample size for birds A) tracked from South Georgia (SG) and B) the Prince Edward Islands (PEI). The 25% and 75% quantiles (shaded areas; SG in red and PEI in blue) and means (black lines) of 1,000 bootstrap iterations are shown. Mkm^2 represents km^2 in millions.

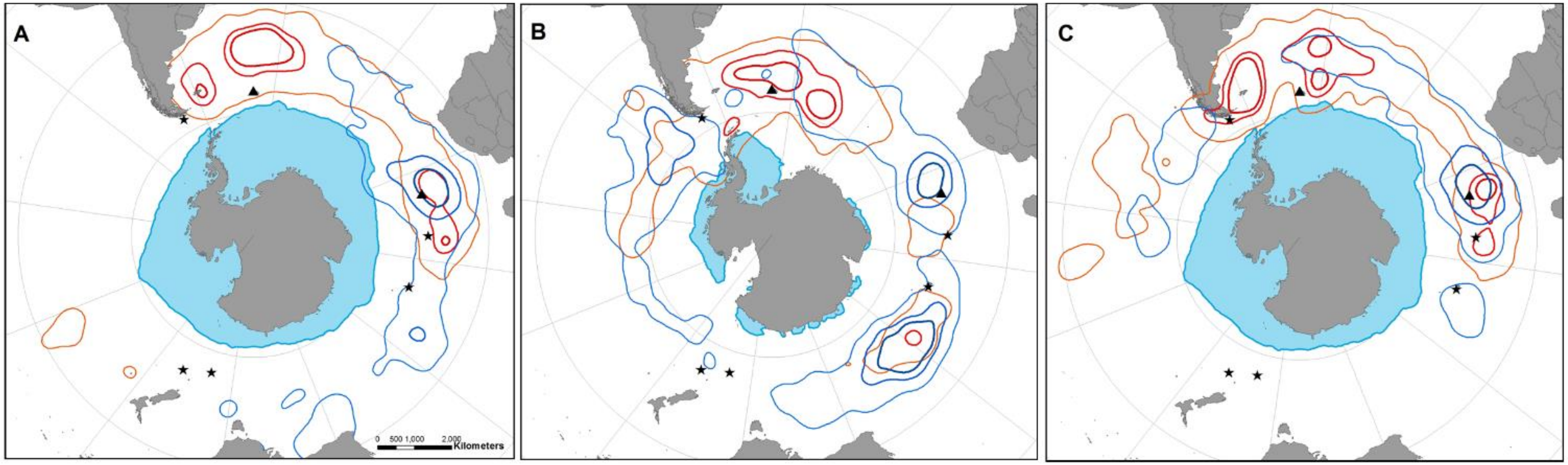

Figure S2. Population-level spatial segregation in non-breeding grey-headed albatross distributions is more pronounced during the non-breeding summer than non-breeding winter. Maps show 25%, 50% and 90% utilization distributions of non-breeding albatrosses from South Georgia (dark blue, blue and light blue) and Prince Edward Islands (dark red, red and orange) in the A) first non-breeding winter (colony departure to mid-September), B) non-breeding summer (mid-September to mid-May) and C) second non-breeding winter (mid-May to colony arrival). Black triangles represent the study colonies and black stars are other breeding colonies. The minimum summer and maximum winter sea ice extents (>15%) are also shown as blue polygons (maps produced by ArcGIS 10.1 software, http://www.arcgis.com/features).

Table S2. Observed and randomized overlap (home-range method, PHR) of core (50%) and general (90%) UDs between different groups of grey-headed albatrosses; population (South Georgia or SG and Prince Edward Islands or PEI) by season, sex by population and season, and breeding outcome by season. Breeding outcome comparisons are for SG only. Randomized overlaps are shown as a mean ± SD and *P* represents the proportion of randomized overlaps that were smaller than the observed. For more information see Supplementary Methods. Significant differences are shown in bold.

Class		50%			90%	
	Observed	Randomized	*P*	Observed	Randomized	*P*
Population (All successful)						
Summer	0.03	0.64 ± 0.07	**<0.001**	0.52	0.82 ± 0.32	**<0.001**
Winter	0.25	0.71 ± 0.09	**<0.001**	0.47	0.78 ± 0.03	**<0.001**
Sex (All successful)						
PEI Summer	0.58	0.60 ± 0.09	0.36	0.74	0.74 ± 0.04	0.42
PEI Winter	0.72	0.81 ± 0.08	0.065	0.69	0.71 ± 0.05	0.26
SG Summer	0.41	0.68 ± 0.09	**0.007**	0.70	0.67 ± 0.05	0.81
SG Winter	0.58	0.56 ± 0.12	0.52	0.75	0.69 ± 0.06	0.88
Breeding status (All SG)						
SG Summer	0.55	0.72 ± 0.06	**0.003**	0.67	0.77 ± 0.04	**0.002**
SG Winter	0.58	0.62 ± 0.10	0.32	0.71	0.71 ± 0.04	0.42

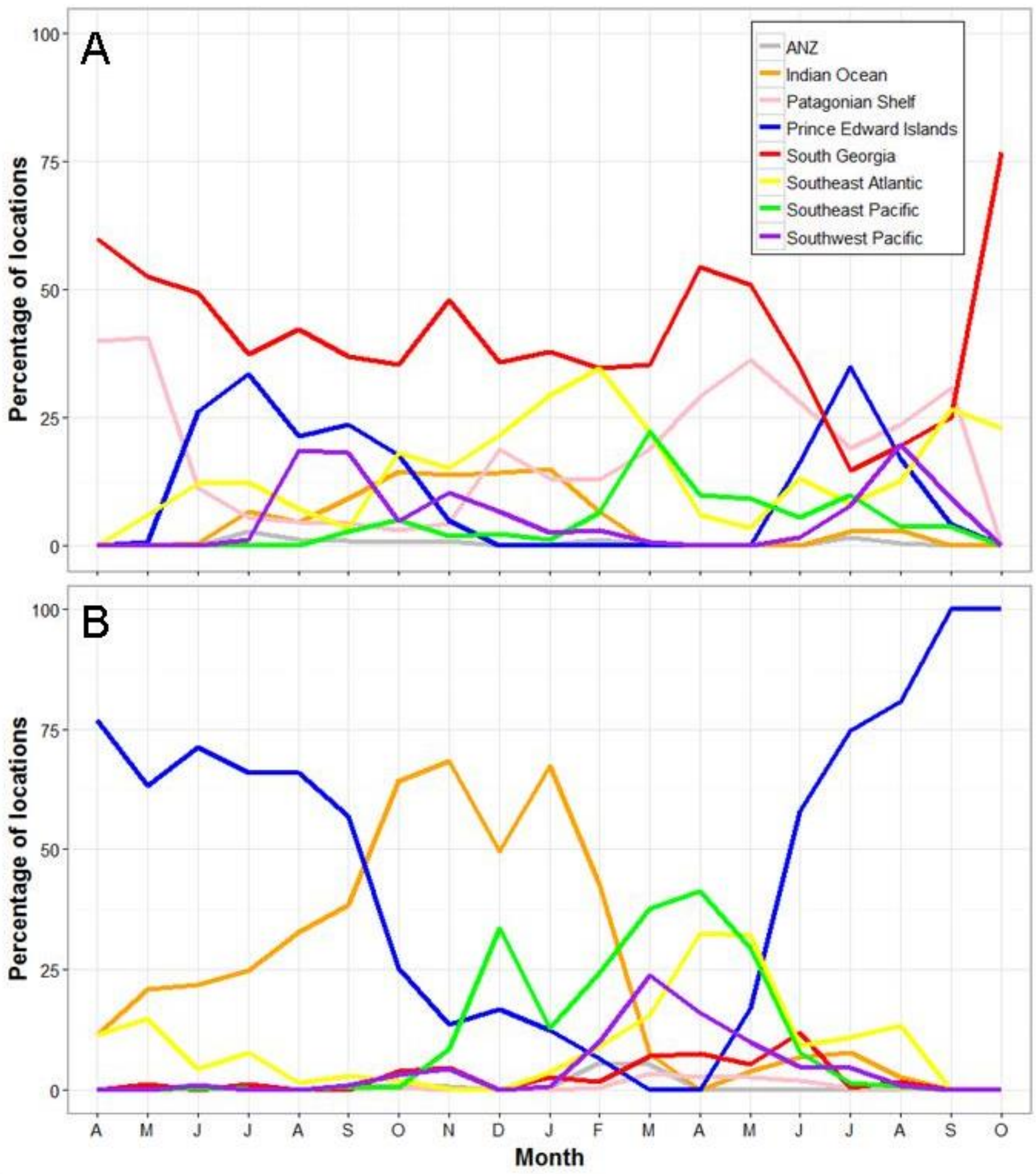

Figure S3. Proportion of successful migratory grey-headed albatrosses from A) South Georgia (SG) and B) Prince Edward Islands (PEI) in different oceanic sectors of the Southern Ocean over the duration of the non-breeding period by month. Notably, birds from both populations largely use different ocean sectors at different times. A large proportion of SG birds (around 40% of locations) remain in the SG area year-round, whereas the PEI birds do not appear to be resident during the summer, when they use the Indian Ocean and Southeast Pacific to a large degree. There is greatest overlap from June to August when the majority of PEI birds and a quarter of SG birds use the PEI region. Months are shown in initials along the

x-axis, starting with April and ending in October the following year. “ANZ” indicates the region encompassing Australia and New Zealand.

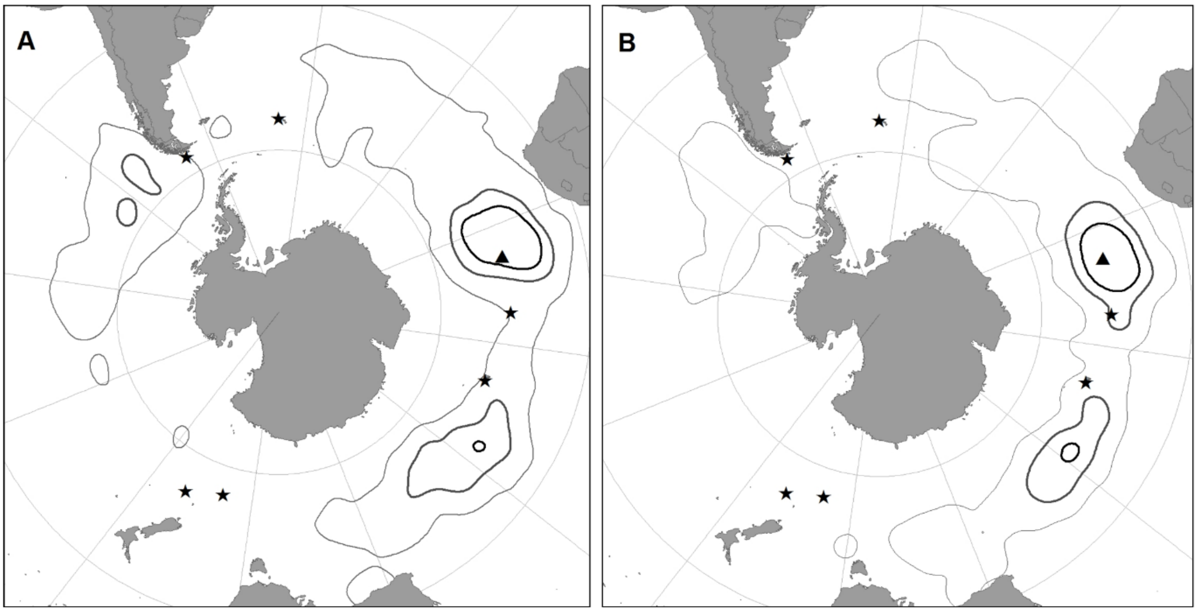

Figure S4. Non-breeding distributions of grey-headed albatrosses from the Prince Edward Islands tracked in A) 2002, and B) 2003. The 25%, 50% and 90% utilization distributions are shown (maps produced by ArcGIS 10.1 software, http://www.arcgis.com/features).

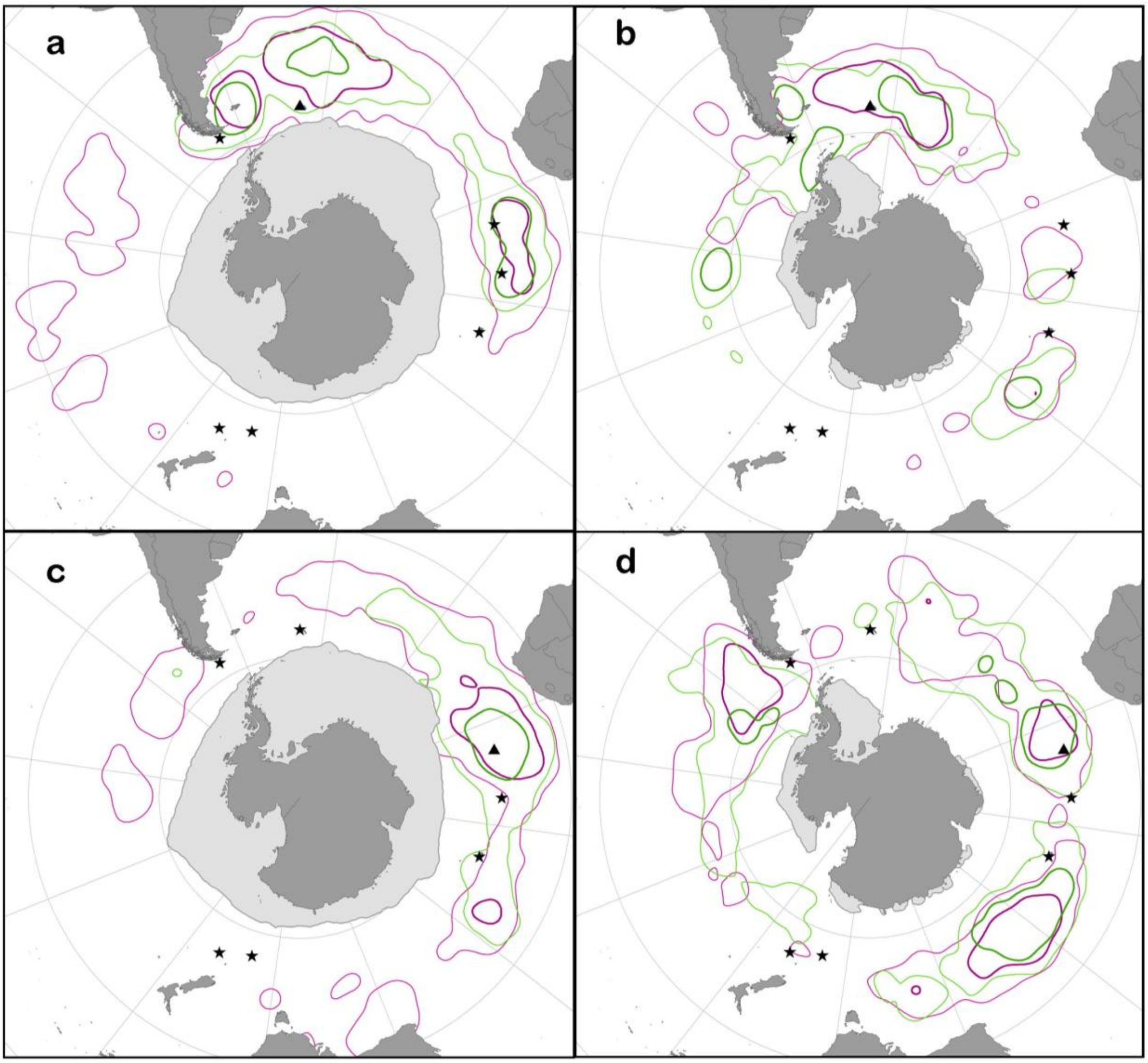

Figure S5. Sex differences in the non-breeding distributions of previously successful grey-headed albatrosses from South Georgia in a) winter and b) summer and Prince Edward Islands in c) winter and d) summer. The 50% and 90% utilization distributions are shown for males in purple and pink, respectively, and for females in dark and light green, respectively. Black triangles represent the study colonies and black stars are other breeding colonies. The minimum summer and maximum winter sea ice extents (>15%) are also shown as light grey polygons (maps produced by ArcGIS 10.1 software, http://www.arcgis.com/features).

Table S3. List of variables used in habitat analysis. Where there is no description, averages were taken of all values within a 200 km buffer.

Variable	Abbreviation	Source	Temporal resolution	Spatial resolution	Description
Bathymetry	*Depth*	GEBCO	-	0.008°	-
Bathymetry gradient	*Depth std*	GEBCO	-	0.008°	Standard deviation of values in 200 km buffer
Sea surface temperature	*SST*	Pathfinder AVHRR *v*.5	Monthly	0.04°	-
Sea surface temperature gradient	*SST std*	Pathfinder AVHRR *v*.5	Monthly	0.04°	Standard deviation of values in 200 km buffer
Chlorophyll a concentration	*Chl*	SeaWiFS from Oceancolor	Monthly	0.08°	-
Eddy kinetic energy	*EKE*	AVISO absolute geostrophic current velocities	8-day	0.25°	-
Sea level anomaly	*SLA*	AVISO delayed-time updated MSLA	8-day	0.25°	-
Wind speed	*Wind*	ERS-2 and QuikSCAT from Ifremer	8-day	0.25°	-
Distance from own colony	*Dist. own*	*Cost distance* tool in ArcGis 10.1	-	-	Custom-generated raster of distance to own colony
Distance from nearest other colony	*Dist. closest*	*Cost distance* tool in ArcGis 10.1	-	-	Custom-generated raster of distance to other colonies

Table S4. Summary of the cross-validation of weekly spatial predictions, showing model performance (AUC) for each predictor type model. Models were constructed to represent the different extrinsic processes (predictor types) that best predict distribution patterns for each GHA population and season. The habitat model contains just environmental predictors, the constraint model contains just distance predictors, and the full model contains all variables. We ran linear models to determine the effect of week and model type on AUCs, and the best model was judged by multi-model inference. For each population and season, AUCs were significantly influenced by predictor type and week, and in each case the best model is shown in bold. For each model means ± SD are for weekly AUC scores with ranges in parentheses.

Model	Full model	Habitat model	Constraint model	Best model	ΔAIC
PEI Summer	**0.772** ± 0.137<br>(0.500 – 0.951)	0.741 ± 0.135<br>(0.501 – 1.000)	0.719 ± 0.122<br>(0.500 – 0.907)	Week +<br>Model type	80.18
PEI Winter	**0.806** ± 0.124<br>(0.511 – 0.988)	0.752 ± 0.109<br>(0.505 – 0.934)	0.772 ± 0.143<br>(0.513 – 0.982)	Week +<br>Model type	45.10
SG Summer	**0.753** ± 0.123<br>(0.501 – 1.000)	0.725 ± 0.101<br>(0.514 – 1.000)	0.703 ± 0.121<br>(0.510 – 1.000)	Week +<br>Model type	34.16
SG Winter	**0.772** ± 0.079<br>(0.533 – 0.938)	0.721 ± 0.059<br>(0.560 – 0.854)	0.702 ± 0.074<br>(0.506 – 0.812)	Week +<br>Model type	33.51

Table S5. Summary of the performance of cross-validation of models with and without breeding outcome differences for South Georgia birds only. We measured model performance using AUC scores at the individual level. Interaction indicates that models included breeding outcome-specific smoothers for each environmental variable and the differences between the AUC scores with and without the interaction for each individual are also shown. Where there are significant differences between AUC scores with and without the interaction, they are emphasized in bold along with the best model. For each model means ± SD are shown with ranges in parentheses.

Model	Interaction	No interaction	Difference	*T test*	*P*
Summer	**0.753** ± 0.110<br>(0.533 – 0.964)	0.726 ± 0.130<br>(0.512 – 0.917)	0.027 ± 0.075<br>(-0.121 – 0.269)	t_{40} = 2.30	**0.027**
Winter	0.753 ± 0.130<br>(0.519 - 0.969)	0.758 ± 0.135<br>(0.508 – 0.961)	-0.005 ± 0.052<br>(-0.159 – 0.107)	t_{40} =-0.59	0.555

Table S6. Summary of the performance of the cross-validation of models with and without sex-differences for successful birds from both populations in summer and winter. We measured model performance using AUC scores at the individual level. Interaction indicates that models include sex-specific smoothers for each environmental variable and the differences between the AUC scores with and without the interaction for each individual are also shown. In each case, there were no significant differences between AUC scores with and without the interaction. For each model, means ± SD are shown with ranges in parentheses.

Model	Interaction	No interaction	Difference	*T test*	*P*
PEI Summer	0.780 ± 0.093<br>(0.510 – 0.892)	0.787 ± 0.081<br>(0.639 – 0.901)	-0.007 ± 0.048<br>(-0.191 – 0.048)	$t_{22} = -0.66$	0.518
PEI Winter	0.804 ± 0.073<br>(0.660 – 0.925)	0.791 ± 0.080<br>(0.660 – 0.916)	0.014 ± 0.046<br>(-0.084 – 0.125)	$t_{23} = 1.46$	0.157
SG Summer	0.763 ± 0.099<br>(0.557 – 0.954)	0.745 ± 0.009<br>(0.554 – 0.929)	0.009 ± 0.052<br>(-0.050 – 0.185)	$t_{20} = -0.79$	0.437
SG Winter	0.758 ± 0.076<br>(0.639 – 0.912)	0.761 ± 0.074<br>(0.648 – 0.904)	-0.003 ± 0.021<br>(-0.048 – 0.039)	$t_{20} = -0.61$	0.549

Table S7. Model selection summaries for birds tracked from PEI and SG during summer and winter. For each model, all combinations were run and ranked based on their parsimony. The five best models are shown.

Model	LogLik	AICc	ΔAICc	Weight
PEI summer				
All variables	-34380.0	68833.67	0.00	1.00
All variables except *SLA*	-34400.0	68869.02	35.35	< 0.01
All variables except *Depth*	-34431.5	68924.60	90.94	< 0.01
All variables except *SST std*	-34436.7	68934.43	100.76	< 0.01
PEI winter				
All variables	-30736.7	61540.93	0.00	0.77
All variables except *Depth std*	-30738.5	61543.31	2.37	0.23
All variables except *SLA*	-30768.1	61592.22	51.29	<0.01
All variables except *EKE*	-30766.8	61593.65	52.71	<0.01
All variables except *Depth std* and *SLA*	-30770.2	61594.80	53.86	<0.01
SG summer				
All variables	-29951.5	59965.50	0.00	0.96
All variables except *SLA*	-29955.4	59972.07	6.57	0.03
All variables except *Wind speed*	-29966.2	59983.49	17.99	<0.01
All variables except *SLA and Wind speed*	-29971.0	59991.16	25.66	<0.01
All variables except *Depth std*	-29968.2	59991.58	26.01	<0.01
SG winter				
All variables	-25573.0	51208.81	0.00	1.00
All variables except *SLA*	-25622.0	51302.88	94.07	< 0.01
All variables except *Depth std*	-25627.6	51309.89	101.08	< 0.01
All variables except *Wind speed*	-25641.9	51340.98	132.17	< 0.01
All variables except *EKE*	-25657.7	51372.55	163.74	< 0.01

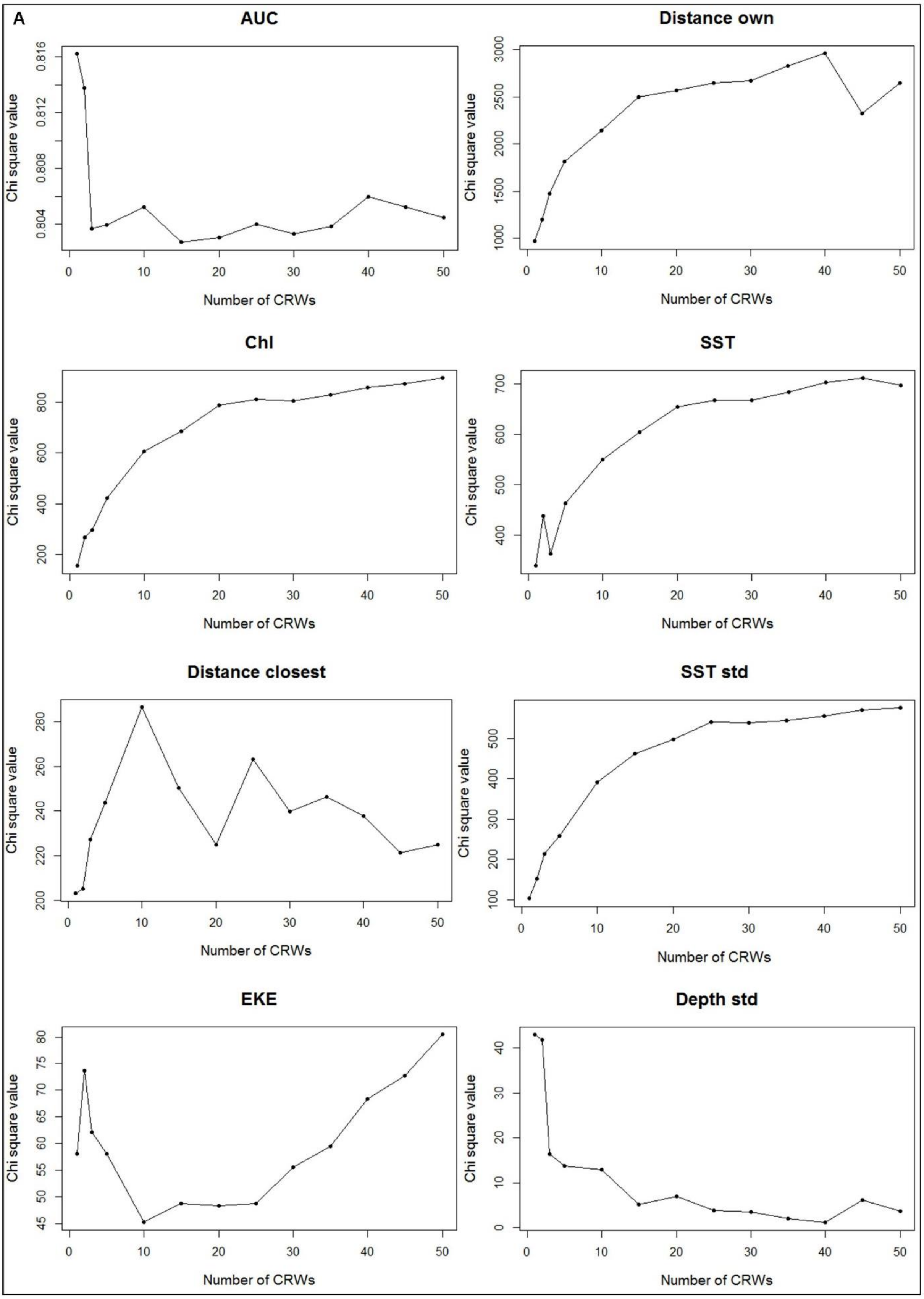
A
AUC
Distance own
Chl
SST
Distance closest
SST std
EKE
Depth std
Chi square value
Number of CRWs

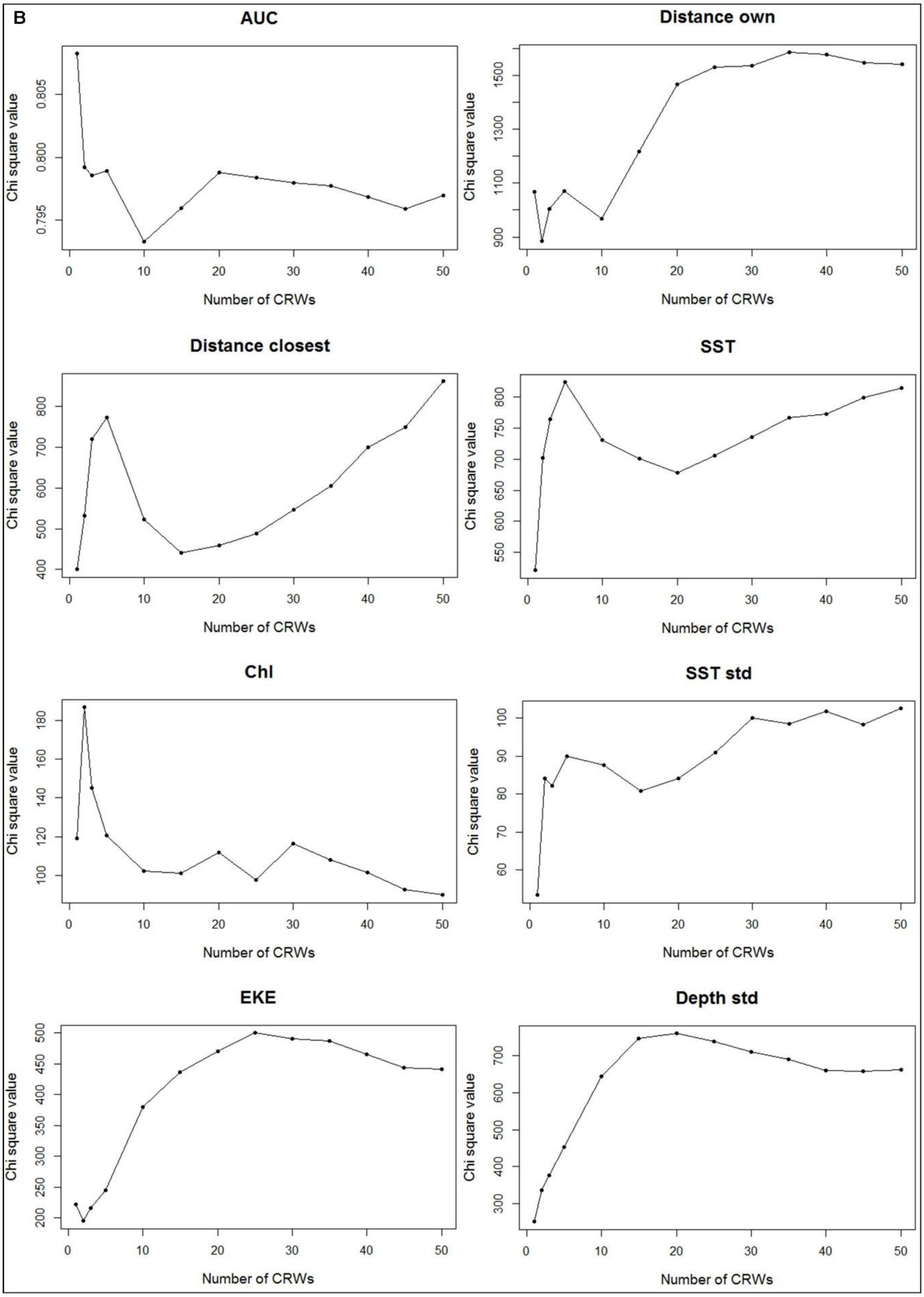

B
AUC
Distance own
Distance closest
SST
Chl
SST std
EKE
Depth std
Chi square value
Number of CRWs

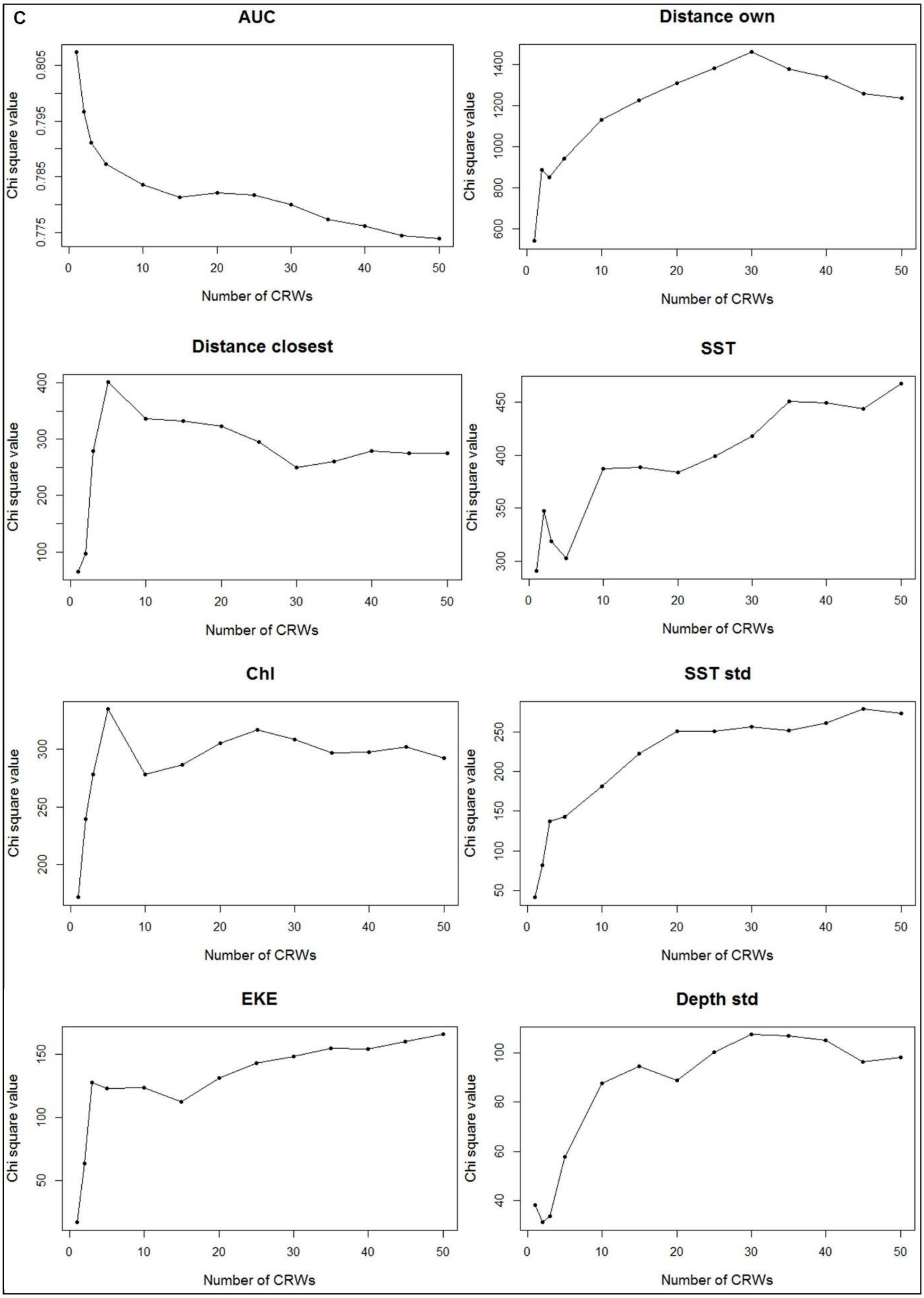
C
AUC
Distance own
Distance closest
SST
Chl
SST std
EKE
Depth std
Chi square value
Number of CRWs

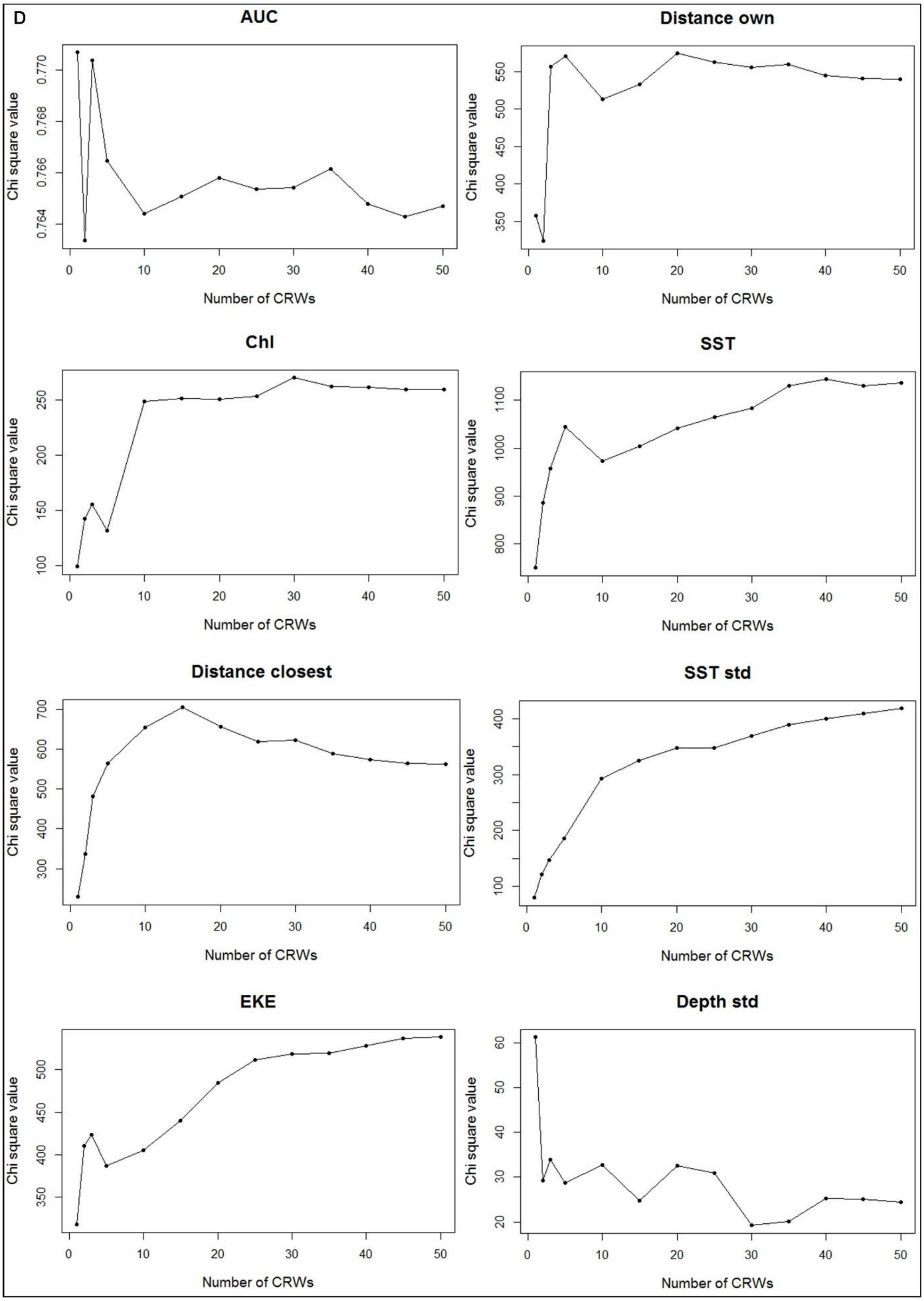

D
AUC
Distance own
Chl
SST
Distance closest
SST std
EKE
Depth std
Chi square value
Number of CRWs

Figure S6. Chi-square values of key predictor variables (explaining >1% deviance explained) and model performance scores (AUC) with increasing number of correlated random walks (CRWs) for A) PEI winter B) PEI summer C) SG winter and D) SG summer models.

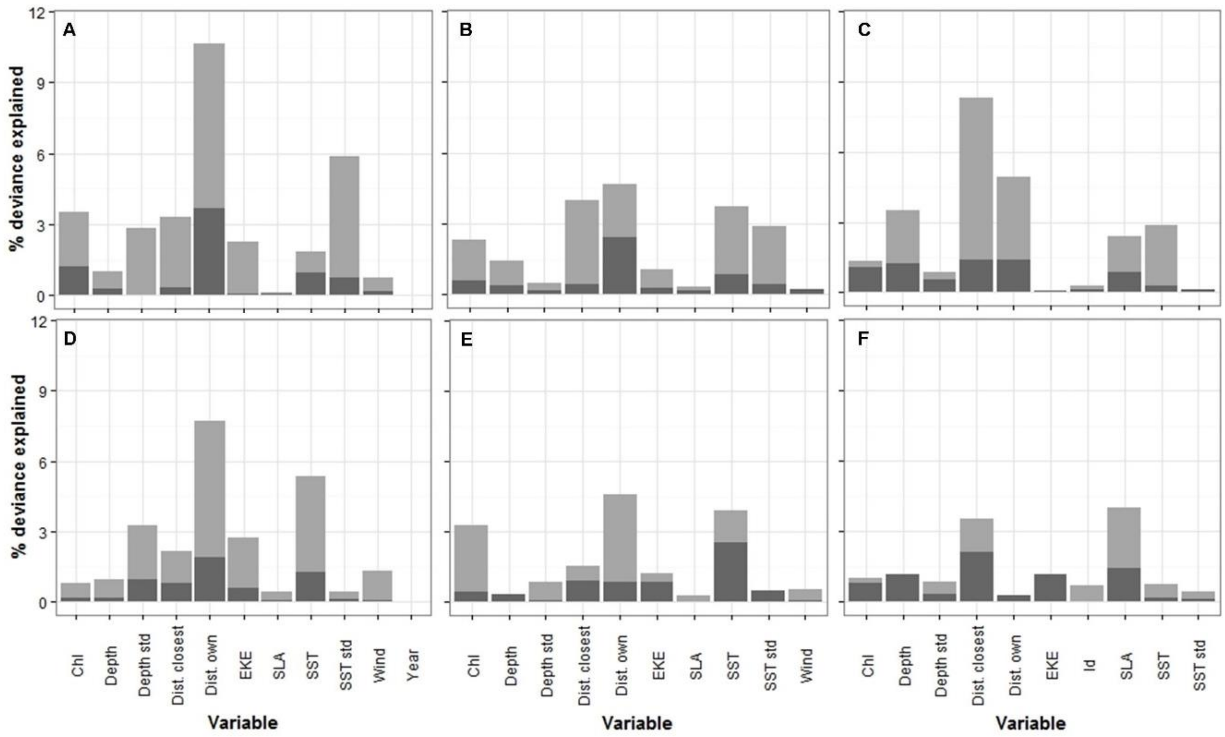

Figure S7. The percentage of deviance explained by important variables (those with >1% deviance explained) for previously successful grey-headed albatrosses for the following models; A) PEI winter, B) SG winter, D) PEI summer and E) SG summer. Also shown are variables for SG failed birds during C) winter and F) summer. Deviance is split into unique deviance explained just by that variable (black), and deviance shared with other variables (grey).

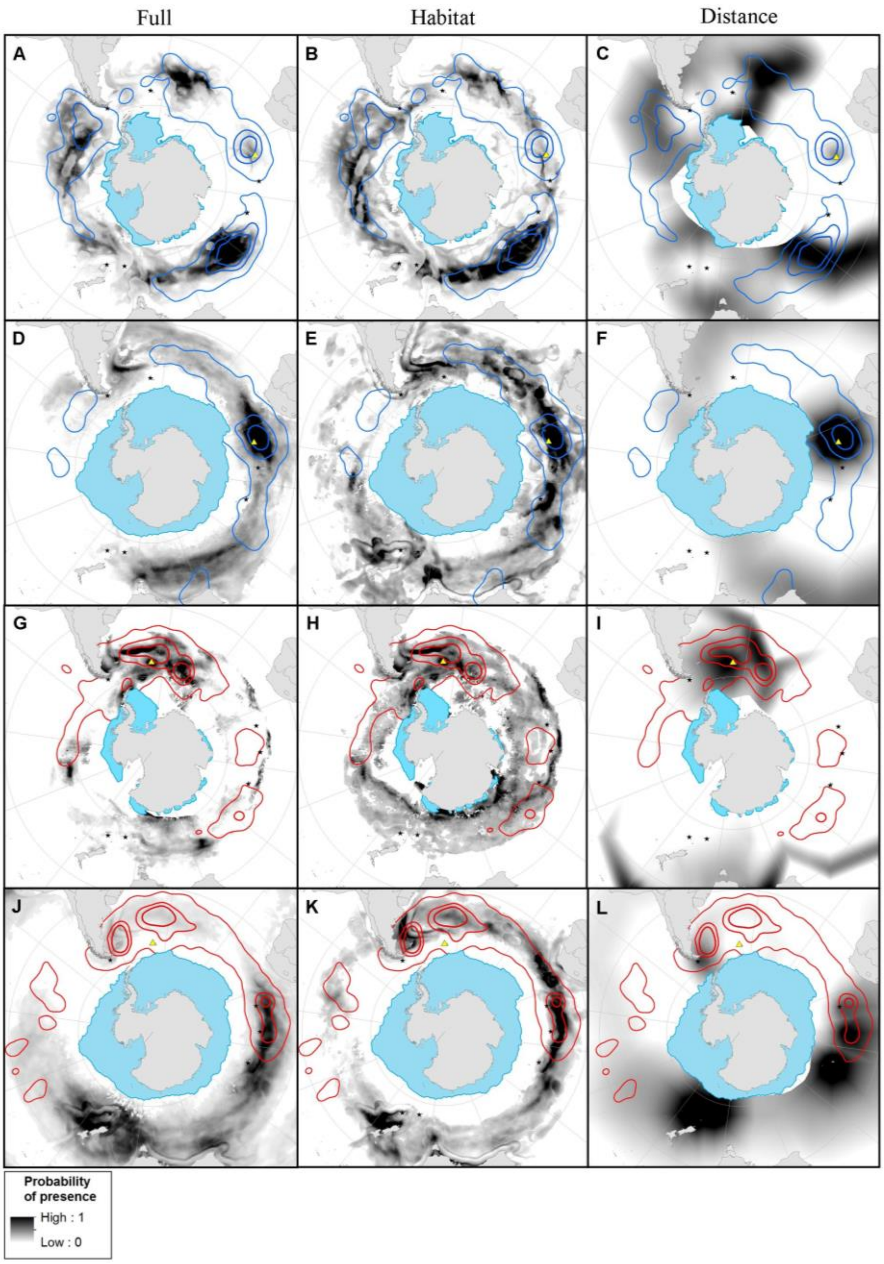

Figure S8. Map of the spatial predictions from the full models (A, D, G, J), those just taking into account habitat preferences (B, E, H, K), and those taking into account accessibility and competition (C, F, I, L). Maps are of suitable habitat from model predictions, scaled to 1 to show probability of presence of birds from different populations in summer and winter; PEI winter (A - C), SG winter (D – F), PEI summer (G – I) and SG summer (J – L). 25%, 50% and 90% kernels of observed distributions are shown for PEI and SG birds in blue or red, respectively. The minimum summer or maximum winter sea ice extents (>15%) are also

shown as blue polygons and the colonies of origin as yellow triangles (map produced by ArcGIS 10.1 software, http://www.arcgis.com/features).
